# Comprehensive transcriptomic analysis provides new insights into the mechanism of ray floret morphogenesis in chrysanthemum

**DOI:** 10.1186/s12864-020-07110-y

**Published:** 2020-10-20

**Authors:** Ya Pu, He Huang, Xiaohui Wen, Chenfei Lu, Bohan Zhang, Xueqi Gu, Shuai Qi, Guangxun Fan, Wenkui Wang, Silan Dai

**Affiliations:** 1 Beijing Advanced Innovation Center for Tree Breeding by Molecular Design, Beijing Key Laboratory of Ornamental Plants Germplasm Innovation & Molecular Breeding, National Engineering Research Center for Floriculture, Beijing Laboratory of Urban and Rural Ecological Environment, Key Laboratory of Genetics and Breeding in Forest Trees and Ornamental Plants of Education Ministry, School of Landscape Architecture, Beijing Forestry University, Beijing, 100083 China; 2 Fuzhou Planning Design & Research Institute, Fuzhou, 350108 China

**Keywords:** Ray floret, Petal morphogenesis, *CYC2s*, Hormone genes, Cell division, Transcriptome analysis

## Abstract

**Background:**

The ray floret shapes referred to as petal types on the chrysanthemum (*Chrysanthemum* × *morifolium* Ramat.) capitulum is extremely abundant, which is one of the most important ornamental traits of chrysanthemum. However, the regulatory mechanisms of different ray floret shapes are still unknown. *C. vestitum* is a major origin species of cultivated chrysanthemum and has flat, spoon, and tubular type of ray florets which are the three basic petal types of chrysanthemum*.* Therefore, it is an ideal model material for studying ray floret morphogenesis in chrysanthemum. Here, using morphological, gene expression and transcriptomic analyses of different ray floret types of *C. vestitum*, we explored the developmental processes and underlying regulatory networks of ray florets.

**Results:**

The formation of the flat type was due to stagnation of its dorsal petal primordium, while the petal primordium of the tubular type had an intact ring shape. Morphological differences between the two ray floret types occurred during the initial stage with vigorous cell division. Analysis of genes related to flower development showed that *CYCLOIDEA* genes, including *CYC2b*, *CYC2d*, *CYC2e*, and *CYC2f,* were differentially expressed in different ray floret types, while the transcriptional levels of others, such as *MADS-box* genes, were not significantly different. Hormone-related genes, including *SMALL AUXIN UPREGULATED RNA* (*SAUR*), *GRETCHEN HAGEN3* (*GH3*), *GIBBERELLIN 2-BETA-DIOXYGENASE 1* (*GA2OX1*) and *APETALA2/ETHYLENE RESPONSIVE FACTOR* (*AP2/ERF*), were identified from 1532 differentially expressed genes (DEGs) in pairwise comparisons among the flat, spoon, and tubular types, with significantly higher expression in the tubular type than that in the flat type and potential involvement in the morphogenesis of different ray floret types.

**Conclusions:**

Our findings, together with the gene interactional relationships reported for *Arabidopsis thaliana*, suggest that hormone-related genes are highly expressed in the tubular type, promoting petal cell division and leading to the formation of a complete ring of the petal primordium. These results provide novel insights into the morphological variation of ray floret of chrysanthemum.

## Background

Colorful and multiform petals are usually the most attractive parts of higher plants. The abundant shape of petals is the breeding goal of many horticulturalists to enhance the ornamental value of plants. Chrysanthemum (*Chrysanthemum* × *morifolium* Ramat.), a valuable ornamental and commercial crop, has a typical radiate capitulum composed of central disc florets and peripheral ray florets and regarded as a pseudanthium [1–3]. Disc florets with an actinomorphic corolla tube are bisexual and fertile, while ray florets are unisexual with various shapes of petals and are usually divided into three basic types including flat, spoon and tubular type according to the corolla tube merged degree (CTMD) which is a morphological index to aid in defining petal type [4]. The diversity of the chrysanthemum capitulum is determined by the relative number and position of disc and ray florets and the petal type of ray florets [5].

A large number of molecular genetics studies have revealed the mechanisms regulating the development of disc florets and ray florets in Asteraceae. Floral organ identity is conferred on developing primordia by the well-characterized ABCE genes, which has been widely confirmed in model plants [6–8]. For Asteraceae, the ABCE genes also regulated the development of the capitulum [8–10]. In *Gerbera hybrida, SEPALLATA*-like MADS box genes, *GERBERA REGULATOR of CAPITULUM DEVELOPMENT* (*GRCDs*), controlled determinacy of the inflorescence meristem [11, 12], and suppression of *GERBERA GLOBOSA-LIKE1* (*GGLO1*), *GERBERA DEFICIENS-LIKE1* (*GDEF1*) and *GDEF2* resulted in retrogressive trans florets [13]. In addition, the relative positions of distinct florets were mainly regulated by an endogenous auxin gradient, the disruption of which led to homeotic conversions of florets and phyllaries in the capitulum [14]. However, the molecular basis of various ray florets, another determinant of the diversity of the capitulum, remains largely unexplored.

The visual difference of three basic ray floret types is the petal symmetry. The flat and spoon types are bilaterally symmetric and tubular types that are approximately radially symmetric. The genetic control of flower symmetry has been deduced in studies of *Antirrhinum majus*, mainly involving *CYC* and its paralog gene *DICHOTOMA* (*DICH*) [15, 16]. In Asteraceae plants, expression level changes or mutations of *CYC2s* significantly affected the morphology of ray florets [17–20]. Because of transposon insertion of *HaCYC2c* in *Helianthus annuus*, the originally zygomorphic flat type of ray floret became the actinomorphic tubular type [21], while overexpression of *RAY2* (*CYC2* homologous gene) in *Senecio vulgaris* resulted in the formation of tubular type [22]. Studies of *C. lavandulifolium*, another ancestral diploid wild species of cultivated chrysanthemum [23], revealed that overexpression of *CYC2c* made the petal of ray floret longer than wild type [24], but ectopic expression of *CYC2d* hindered the growth of ray floret petals [25]. Previous studies have shown that the functions of *CYC2* genes in ray florets of Asteraceae plants were quite different, which could not explain the reason for the formation of different ray floret types.

The growth process of petals in higher plants mainly involves three stages: ① petal primordium initiation; ② petal cell proliferation in the early stage; and ③ petal cell expansion in the late stage [26, 27]. Auxin has been shown to directly signal the initiation of petal primordia, and mutations in genes related to auxin biosynthesis, transport, and response all dramatically affect petal formation [28, 29]. The morphological difference in petals may appear in the early stage of vigorous cell division or in the late stage, when cell expansion predominates over cell division [30]. The proliferation and expansion of petal cells are significantly affected by plant hormones. In Arabidopsis, *AP2/ERF* regulated by auxin-related genes [31–33] promoted cell proliferation in the early phase of petal growth [34, 35]. In addition, plant hormones, including auxin, cytokinin (CTK), gibberellin (GA), abscisic acid (ABA), and brassinolide (BR), also affected petal cell expansion of ray florets in Asteraceae [36–38]. In gerbera, GhWIP2, a WIP zinc finger protein, acted as a transcriptional repressor to suppress cell expansion and affect the final morphology of ray florets by regulating the levels of GA, ABA, and auxin [38]. For chrysanthemum, however, the stage in which the morphological difference in different ray florets appears and the genes involved in regulating such morphological differences remain unknown.

An extremely rich ray floret shape is a determinant of various chrysanthemum capitulum morphologies. Simultaneously, ray floret shape is an important basis for chrysanthemum cultivar classification [39, 40]. However, the mechanism underlying the formation of different ray floret types is still unclear, and there is a lack of morphological observation and molecular biological exploration. It is difficult to explore these mechanisms because of the extremely abundant morphological variation of ray florets and excessively complex genetic background in chrysanthemum. As an ancestral wild species of chrysanthemum [41], *C. vestitum* is distributed in the high mountain region of central China and has basic ray floret shapes of the flat, spoon and tubular type [42, 43], so it is considered as an important model for studying ray floret morphogenesis. In the current study, phenotypic observation, gene expression analysis and transcriptome sequencing were conducted to explore the morphological nature of ray floret and excavate key genes regulating the ray floret types of *C. vestitum*. Our research not only provides new insights into the development of different ray floret types but also lays the theoretical foundation for directional breeding of flower type in chrysanthemum.

## Results

### Phenotypic observation of different types of capitula and ray florets

**Fig. 1:**
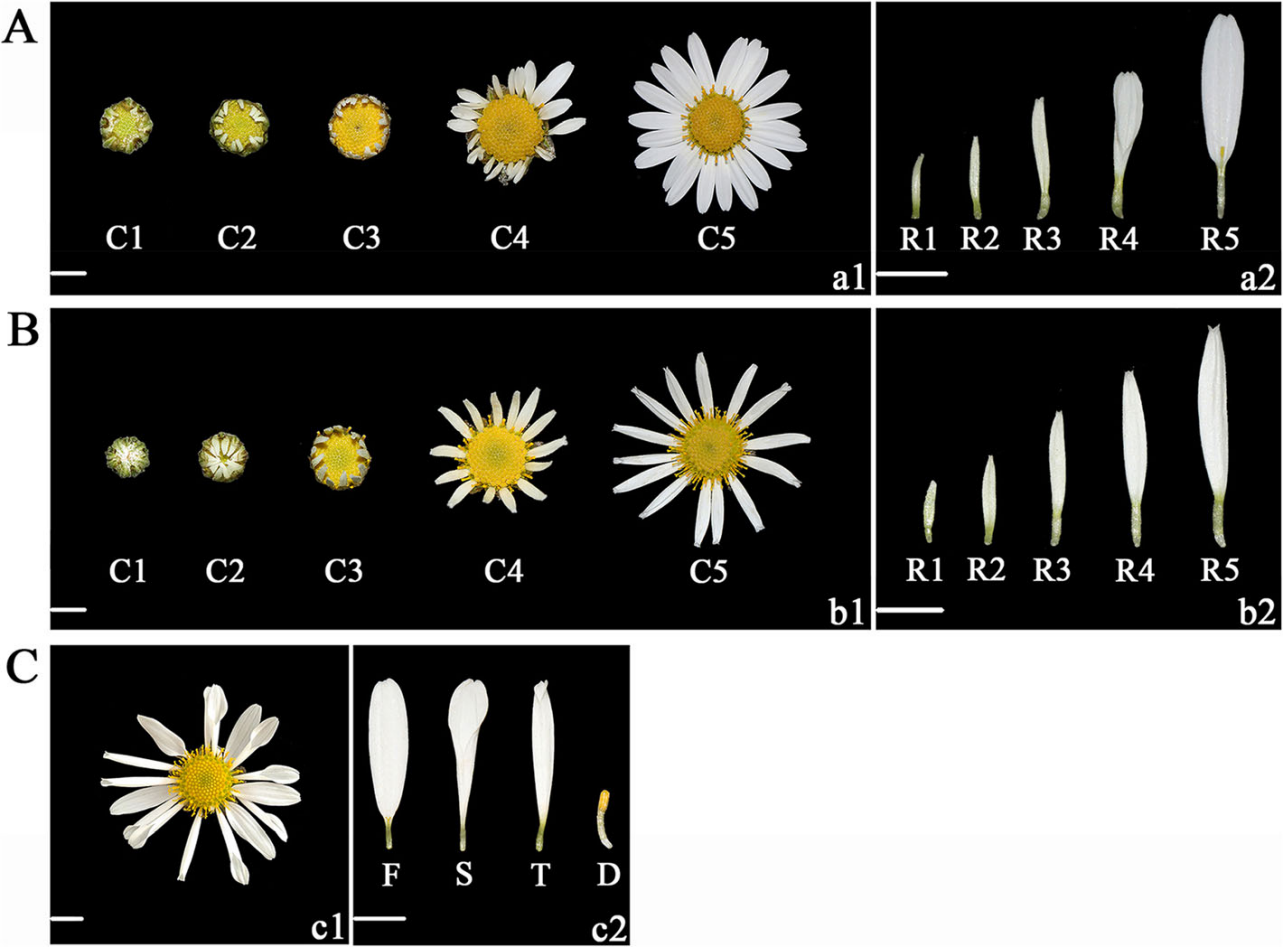
Characterization of three *Chrysanthemum vestitum* strains. **A** The five different opening stages of the CVW capitula (**a1**) and ray florets (**a2**). **B** The five different opening stages of the CVT capitula (**b1**) and ray florets (**b2**). **C** The last opening stage of the CVW capitulum (**c1**), ray florets and disc floret (**c2**). C: capitulum, R: ray floret, F: flat type, S: spoon type, T: tubular type, D: disc floret. Scale bar = 0.5 cm

**Fig. 2:**
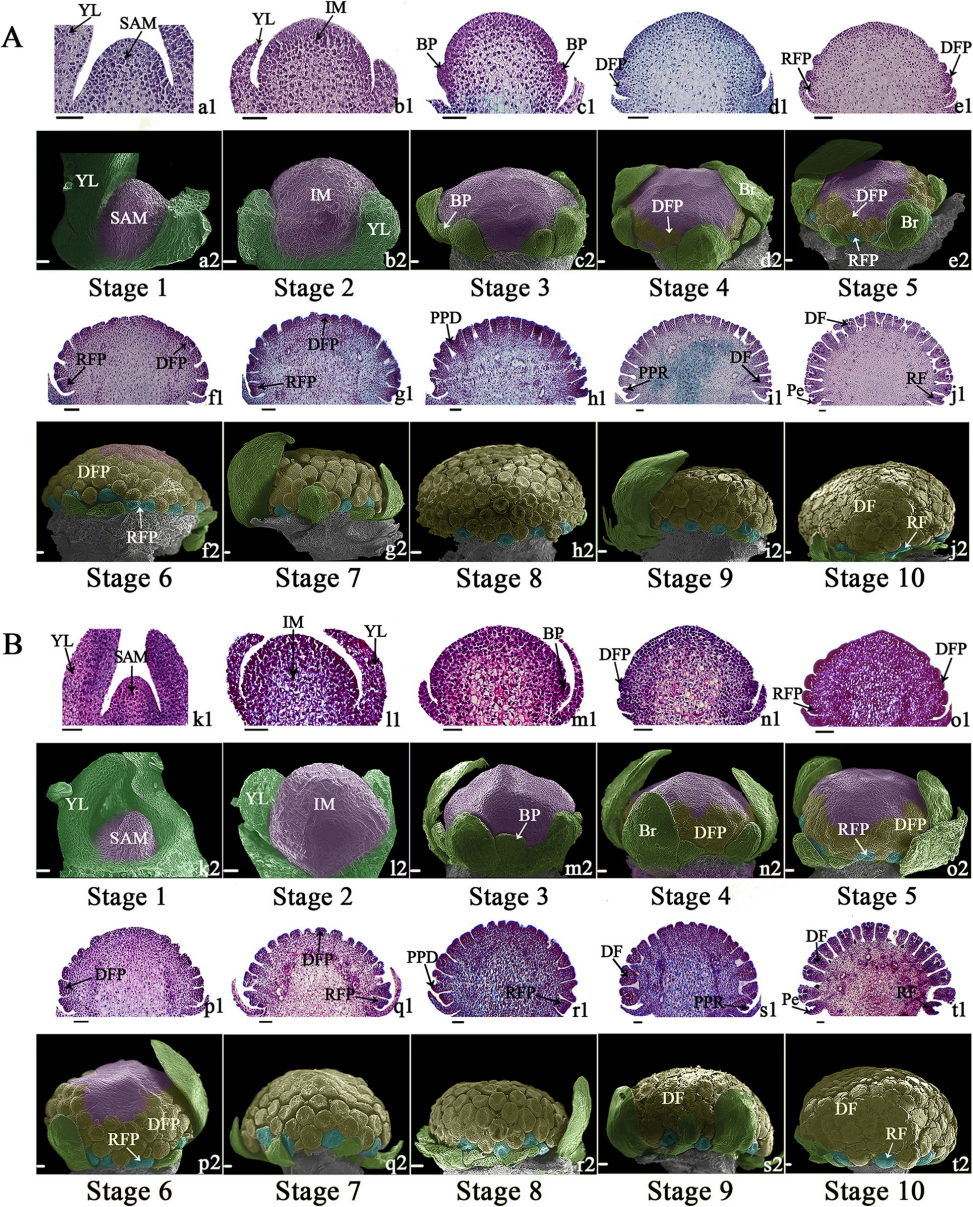
The developmental process of capitulum morphogenesis in *C. vestitum*. **A** The different developmental stages of the CVW capitulum. **B** The different developmental stages of the CVT capitulum. **a1-t1** The capitula showed with paraffin section under optical microscope, scale bar = 500 μm at stage 1–10. **a2-t2** The capitula under scanning electron microscope, scale bar = 200 μm at stage 1–7 and scale bar = 500 μm at stage 8–10. SAM: shoot apical meristem, IM: inflorescence shoot apical meristem, YL: young leaf, BP: bracts primordia, DFP: disc floret primordia, RFP: ray floret primordia, Br: bracts, PPD: petal primordia of disc floret, PPR: petal primordia of ray floret, DF: disc floret, RF: ray floret, Pe: petal

Various plant lines of *C. vestitum* with different ray floret types were collected. Among these plant lines, the ray florets of CVW are all flat type (Fig. 1a), those of CVT are all tubular type (Fig. 1b), and CVZ has three ray floret shapes including flat, spoon and tubular type (Fig. 1c). To determine the key period of phenotypic differences in different types of ray florets, morphological observation was performed of capitula and ray florets of CVW and CVT using paraffin sections and scanning electron microscopy (SEM). Capitulum morphogenesis was divided into ten stages (Fig. 2) based on landmarks (Table 1). When the capitulum had developed to stage 5 (Fig. 2e1, e2, o1, o2), ray floret primordia (RFP) initiated between the bracts and the outermost disc floret primordia (DFP). RFP appeared after one or two rows of DFP formation at stage 4 (Fig. 2d1, d2, n1, n2), which revealed that the floret events on the *C. vestitum* capitulum took place in a non-acropetal or non-centripetal sequence. Comparing the dynamic developmental processes of CVW (Fig. 2a) and CVT (Fig. 2b), we found no difference in the initial time and location between different ray floret types, and the overall developmental processes of CVW and CVT capitula were basically the same.

**Table 1:** A schedule for capitulum morphogenesis and development stages of *C. vestitum*

Stage no.	Stage name	Landmarks of morphological
Stage 1	Vegetative period	SAM keeps the conical shape and is wrapped tightly by young leaves
Stage 2	Apical meristem enlargement stage	Apical meristem grows and expands, showing hemispherical shape and developing into IM
Stage 3	Bract formation stage	Bract primordia start to from at the basal part of IM
Stage 4	Disc floret primordia formation early stage	Disc floret primordia showing as small spherical protrusions initiate at the lower part of IM
Stage 5	Ray floret primordia formation early stage	Ray floret primordia showing as approximate elliptical protrusions initiate between the bract and the outermost disc floret primordia
Stage 6	Floret primordia formation middle stage	Disc floret primordia continue to generate in centripetal differentiated pattern
Stage 7	Floret primordia formation end stage	Floret primordia cover the entire dome of capitulum
Stage 8	Petal formation early stage	The petal primordia of florets begin to form
Stage 9	Petal formation middle stage	Disc floret petals have basically formed, and ray floret petals continue to develop
Stage 10	Petal formation end stage	Disc floret petals are mature and ray floret petals continue to elongate

**Fig. 3:**
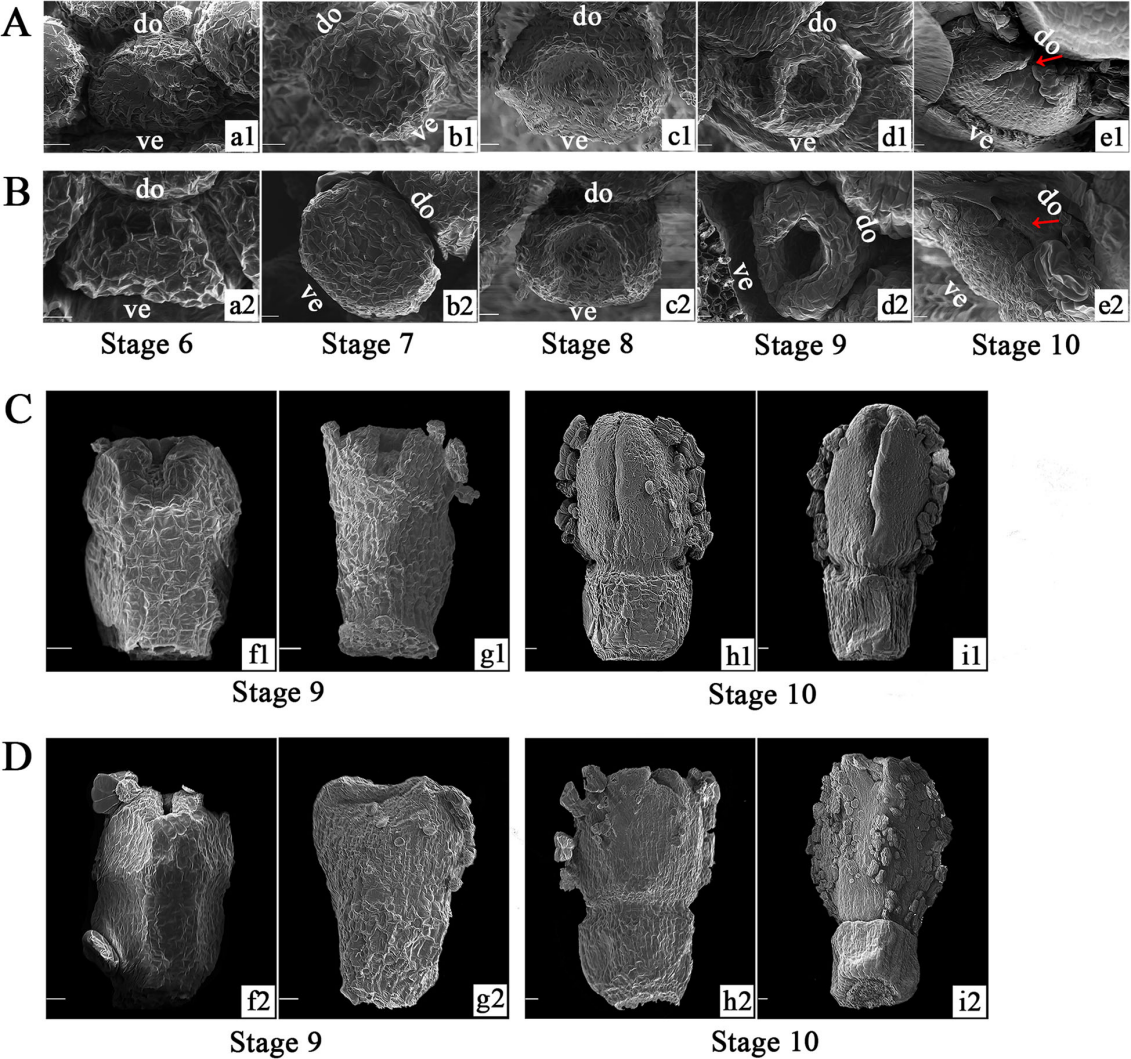
Developmental process of different ray florets types in *C. vestitum* under scanning electron microscope. **A** Top views of CVW ray florets at stage 6–10 (**a1-e1**), scale bar = 100 μm. **B** Top views of CVT ray florets at stage 6–10 (**a2-e2**), scale bar = 100 μm. **C** Developmental process of CVW ray florets on the dorsal at stage 9–10 (**f1-i1**), scale bar = 200 μm. **D** Developmental process of CVT ray florets on the dorsal at stage 9–10 (**f2-i2**), scale bar = 200 μm. ve: ventral, do: dorsal

On the basis of determining the developmental process of the CVW and CVT capitulum, the different types of ray florets morphogenesis were further observed. There was no significant difference in phenotype between the ray florets of CVW and CVT from stage 6 to stage 8 (Fig. 3a1-c1, a2-c2). The initiation of ray floret development was the oval or nearly oval RFP formation, and then the center of RFP sagged inward to present a cup-like structure at stage 7 (Fig. 3b1, b2). At stage 8 (Fig. 3c1, c2), two petal primordium developed on both sides of the cup-shaped structure and gradually grew at stage 9 (Fig. 3d1, d2). The differences between CVW and CVT ray floret morphology were already present at stage 10 (Fig. 3e1, e2). During stage 9 to stage 10 (Fig. 3c, d), petal cell division was vigorous, and the growth of the CVW ray floret dorsal petal stagnated, while the ventral petal quickly elongated and wrapped from both sides to the dorsal. Eventually a fissure presented on the dorsal, resulting in formation of flat ray floret (Fig. 3c). The petals on the ventral and dorsal of CVT grew normally, eventually forming the tubular type (Fig. 3d).

**Fig. 4:**
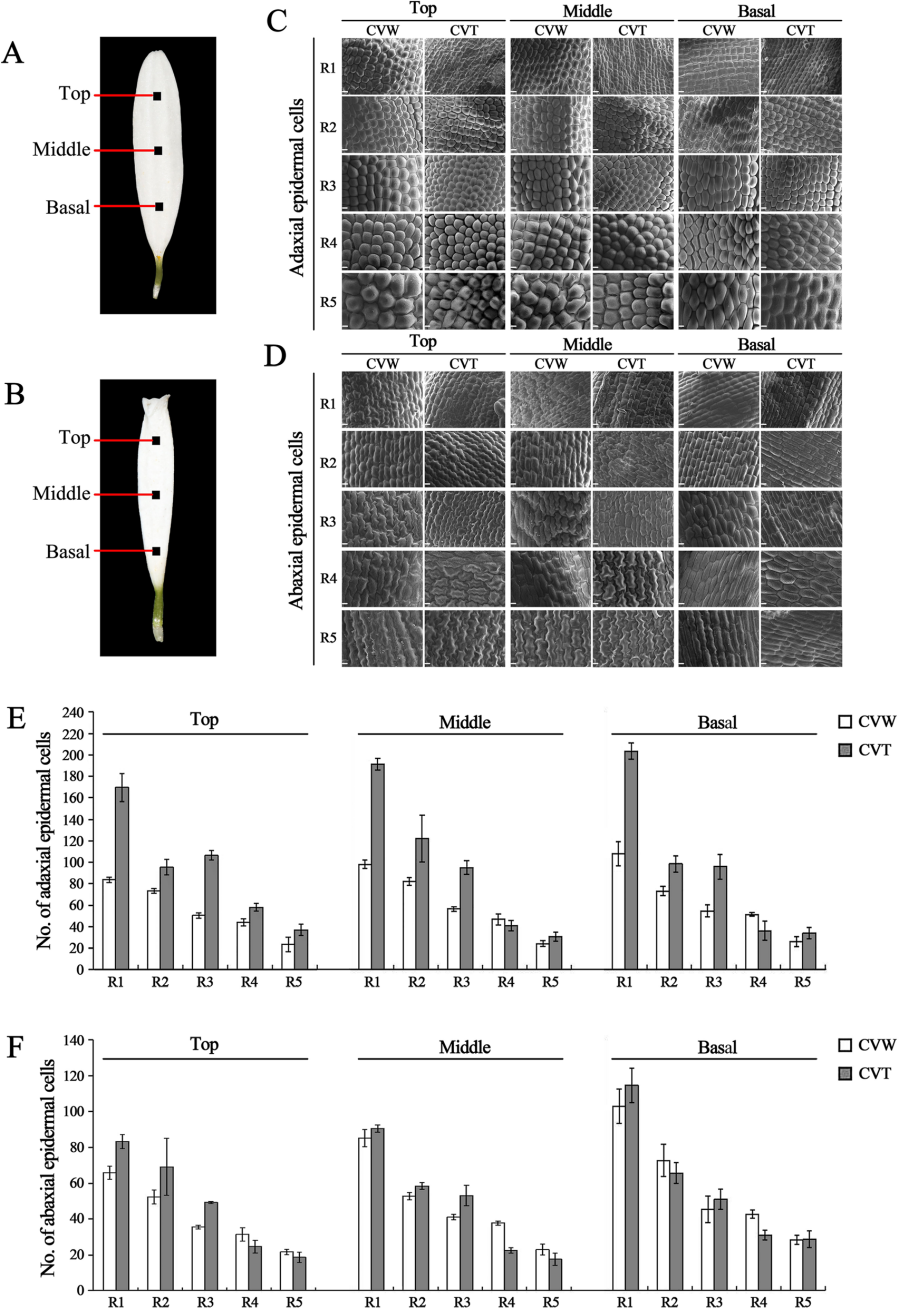
Observation and statistics of CVW and CVT petal epidermal cells of ray floret . **a-b** The materials from the center of the top, middle and basal regions of CVW (**a**) and CVT (**b**) ray florets were sampled for morphological characterization of petal epidermal cells. **c-d** Epidermal cells in the top, middle and basal regions of CVW and CVT ray floret petals were observed using a scanning electron microscope. Scale bar = 100 μm. **e** Measurement of adaxial epidermal cell numbers in CVW and CVT ray floret petals. **f** Measurement of abaxial epidermal cell numbers in CVW and CVT ray floret petals

Based on the observations of the adaxial and abaxial epidermal cells at the center of the basal, middle and top regions of CVW and CVT ray floret petals (Fig. 4a, b) at R1-R5 stage, there was no significant difference between the adaxial and abaxial epidermal cells in terms of morphology (Fig. 4c, d). The number of adaxial epidermal cells in the top, middle, and basal parts of ray floret petals at R1 stage in CVT was observably larger than in CVW at the same magnification. As the capitulum gradually opened, the gap in the number of adaxial epidermal cells between the two narrowed (Fig. 4e), while for the abaxial epidermal cells, there was only a small gap between the number of CVW and CVT in the top, middle, and basal parts at R1-R5 stage (Fig. 4f). The results revealed a major difference in the number of petal epidermal cells between flat and tubular ray floret, and the number of epidermal cells in the tubular type was significantly higher than the flat type.

### Expression pattern of flower development related-genes in different ray floret types

**Fig. 5:**
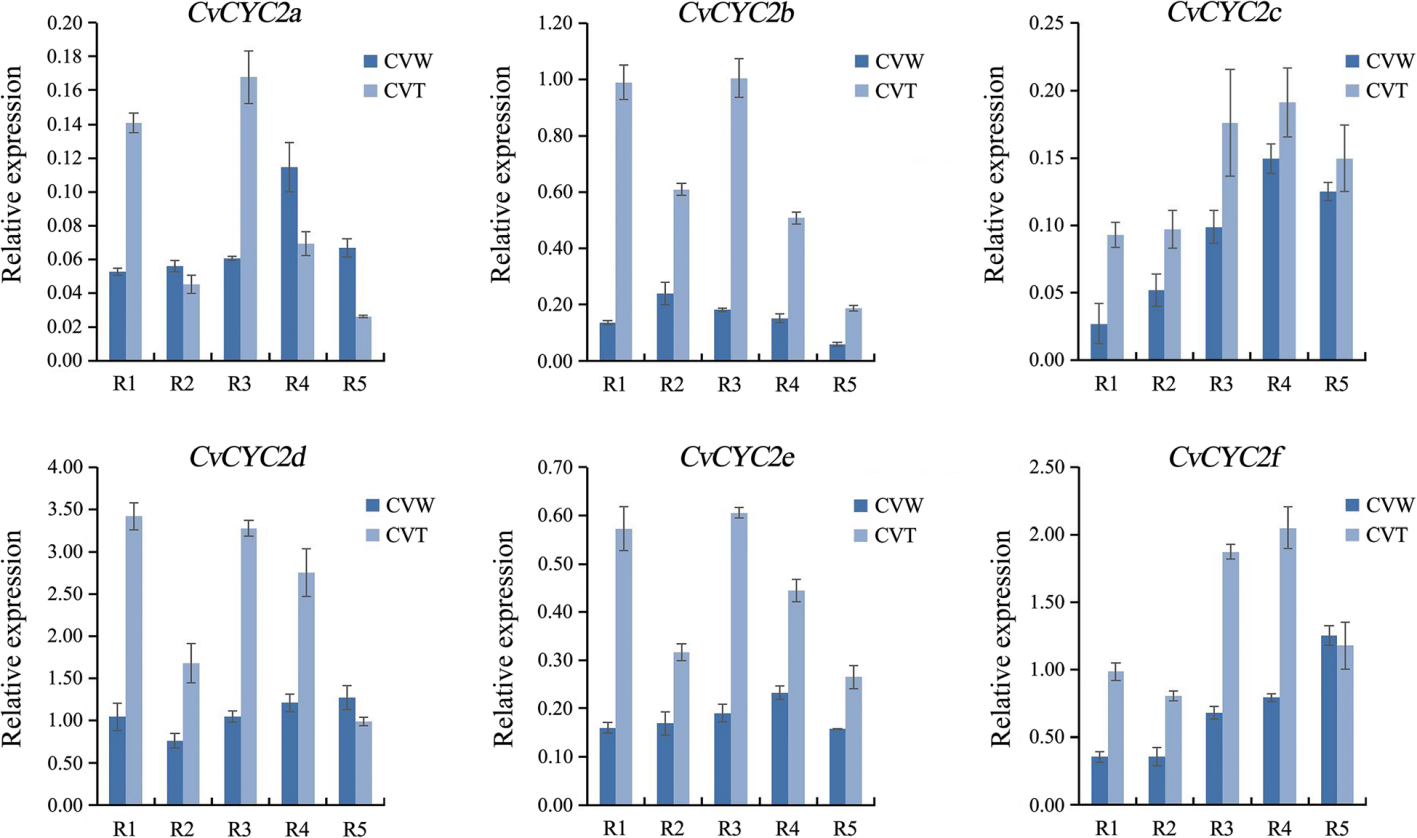
Expression analysis of *CYC2*-like genes in ray floret petals of CVW and CVT. R1-R5 indicated the five opening stages of ray floret

The expression pattern of genes related to flower development, including *MADS-box* conferring floral organ identity, *TEOSINTE BRANCHED/CYCLOIDEA/PCF* (*TCP*) affecting flower symmetry, *NAM/ATAF/CUC* (*NAC*) regulating organ boundaries, WOX effecting petal fusion and *AUXIN RESPONSE FACTOR* (*ARF*), were analyzed in CVW, CVT and CVZ using semi-quantitative reverse transcriptase-polymerase chain reaction (RT-PCR). The expression levels of *CYC2b* and *CYC2e* were higher in CVT than CVW. However, the *MAD-box*, *TCP*, *WOX* and *NAC* genes showed no significantly differential expression among the different samples (Additional file 1: Fig. S1, Additional file 2: Fig. S2). According to further analysis of the expression pattern of *CYC2*-like genes in ray floret petals of CVW and CVT at R1-R5 stage by real-time quantitative polymerase chain reaction (qRT-PCR) (Fig. 5), we found that *CvCYC2b* and *CvCYC2e* lower expression level in CVW than CVT. The expression level of *CvCYC2a* showed no obvious difference between the flat and tubular types, and the expression levels of *CvCYC2c* were slightly different in the two types. *CvCYC2d* and *CvCYC2f* had higher expression levels in CVT than CVW at R1-R4 stage but a similar expression level at R5 stage. The above results suggested that *CvCYC2b*, *CvCYC2d*, *CvCYC2e*, and *CvCYC2f* were important for ray floret morphogenesis.

### Transcriptome sequencing and functional annotation

Because the flat, spoon and tubular ray florets of CVZ were on the same capitulum with the same genetic background, RNA-seq of these samples was carried out to further investigate the molecular mechanisms underlying the ray floret phenotype. The use of three biological repeats resulted in the sequencing of a total of 9 RNA samples (Additional file 3: Fig. S3, Additional file 4: Table S1). A total of 70.79 Gb clean data were generated, and 92.63–97.29% of the clean reads had Phred-like quality scores at the Q30 threshold (percentage of sequences with sequencing error rates lower than 0.1%). Following assembly, 100,882 unigenes were recognized, of which 21,315 were longer than 1 kb and the N50 of the unigenes was 1251 bp. A total of 48,662 unigenes were annotated based on BLASTx (E-value < 1 × 10^− 5^) and HMMER (E-value < 1 × 10^− 10^) searches against public databases including COG, GO, KEGG, KOG, Pfam, Swiss-Prot, eggNOG and Nr. Based on the annotation results (Additional file 5: Fig. S4), 26,253 genes (53.95%) were annotated in KOG, 30,992 genes (63.69%) in Pfam, 29,766 genes (61.17%) in Swiss-Prot, 42,792 genes (87.94%) in eggNOG and 45,723 genes (93.96%) in Nr. The functions of the predicted unigenes were classified using GO, COG, and KEGG assignments. A total of 29,582 genes (60.79%) were annotated by GO assignments, being categorized into three major groups (cellular component, molecular function, and biological process). In addition, 13,290 genes (27.31%) were clustered into 25 COG categories, and 17,764 genes (36.50%) were mapped into 129 KEGG pathways, with the most represented pathways being “Ribosome (ko03010)” followed by “Carbon metabolism (ko01200)”.

### DEGs identified by pairwise comparison

**Fig. 6:**
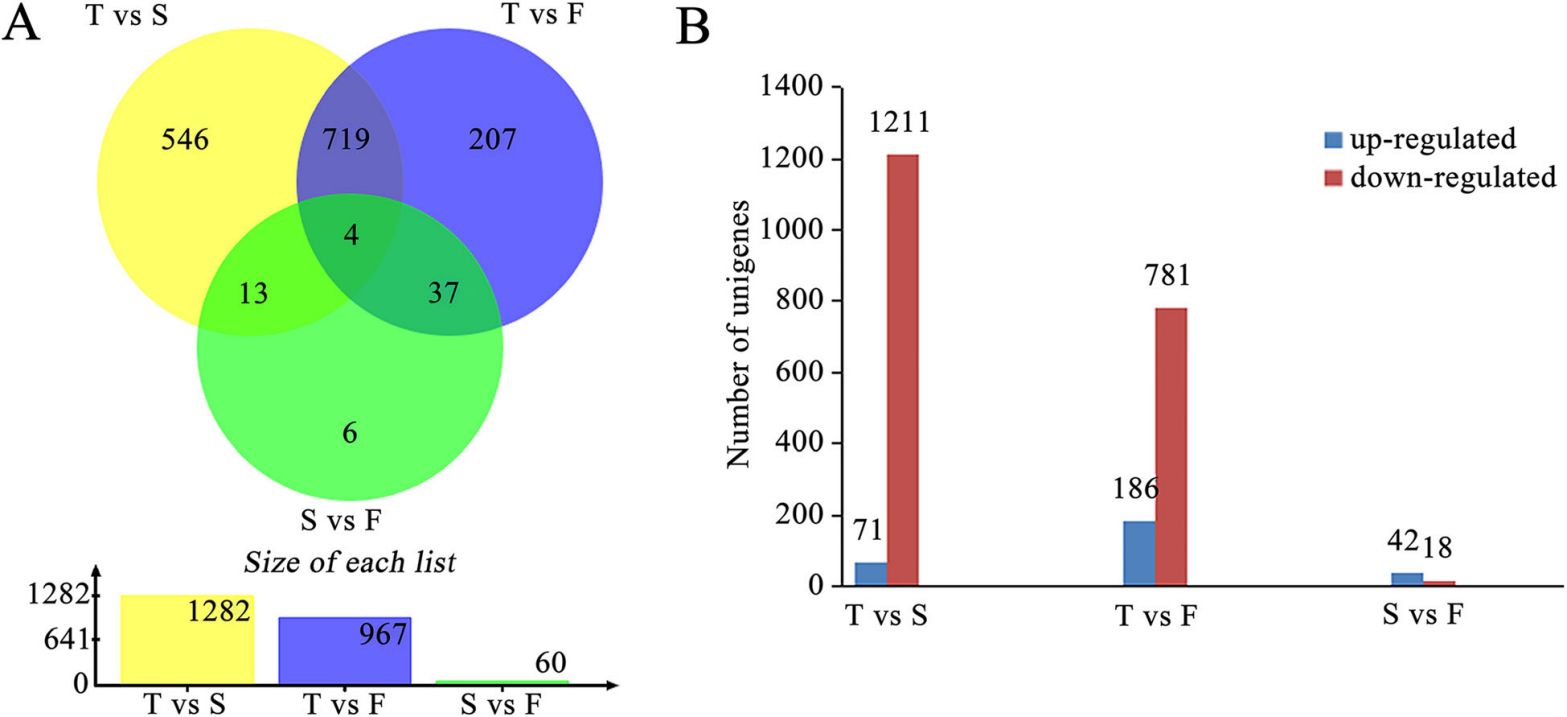
DEGs among the flat, spoon and tubular types of CVZ. **a** Venn diagram showing the number of DEGs revealed by paired comparison. **b** The number of up-regulated and down-regulated DEGs in comparisons among T_vs_S, T_vs_F and S_vs_F

To identify the important genes associated with ray floret morphogenesis, a pairwise comparison was conducted among the flat, spoon and tubular types. A total of 1532 DEGs were detected in the three comparisons, with 1282 (71 up-regulated and 1211 down-regulated), 976 (186 up-regulated and 781 down-regulated), and 60 (42 up-regulated and 18 down-regulated) DEGs in T_vs_S, T_vs_F and S_vs_F, respectively (Fig. 6a, b). Only 4 DEGs were present in all three comparisons (Additional file 6: Table S2). Furthermore, the overlapping number of DEGs detected from T_vs_S and T_vs_F was 719, from T_vs_F and S_vs_F was 37 and from T_vs_S and S_vs_F was 13 (Additional file 6: Table S2), which might contain important factors affecting the shape of ray florets. To classify the functions of DEGs in pairwise comparisons, they were further evaluated using GO and KEGG. The GO enrichment of DEGs presented a similar pattern compared with all genes (Additional file 7: Fig. S5A-C). The most enriched pathway in DEGs of T_vs_S and T_vs_F was “phenylpropanoid biosynthesis (ko00940)” (Additional file 7: Fig. S5D-F).

**Fig. 7:**
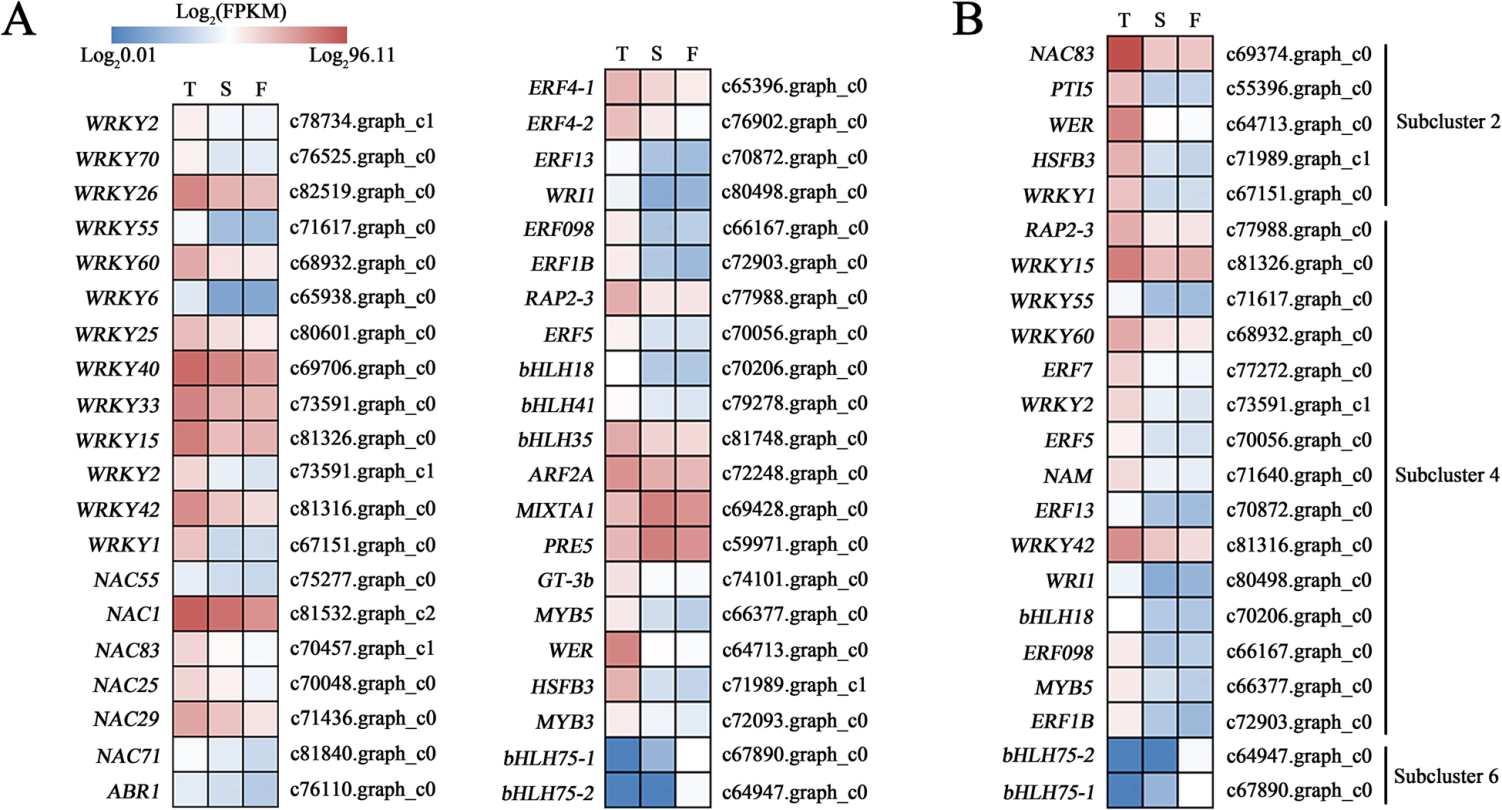
Heatmap representation of the expression patterns of transcription factors in T (tubular type), S (spoon type) and T (flat type) of CVZ. **a-b** Transcription factors detected by pairwise comparison (**a**) and K-means cluster analysis (**b**)

Transcription factors (TFs) play important roles in the regulation of flower organ morphogenesis. There were 39 TFs in the overlapping DEGs of T_vs_S and T_vs_F, mainly including WRKY, NAC, ARF, AP2/ERF, basic helix-loop-helix (bHLH). Only 2 genes were predicted to encode transcription factors in the overlapping DEGs of T_vs_F and S_vs_F, both of which were bHLH. The 41 genes encoding transcription factors were differentially expressed in different types of ray florets, most of which were expressed at the highest levels in the tubular type and at lower levels in the flat type (Fig. 7a).

### Cluster analysis of DEGs

**Fig. 8:**
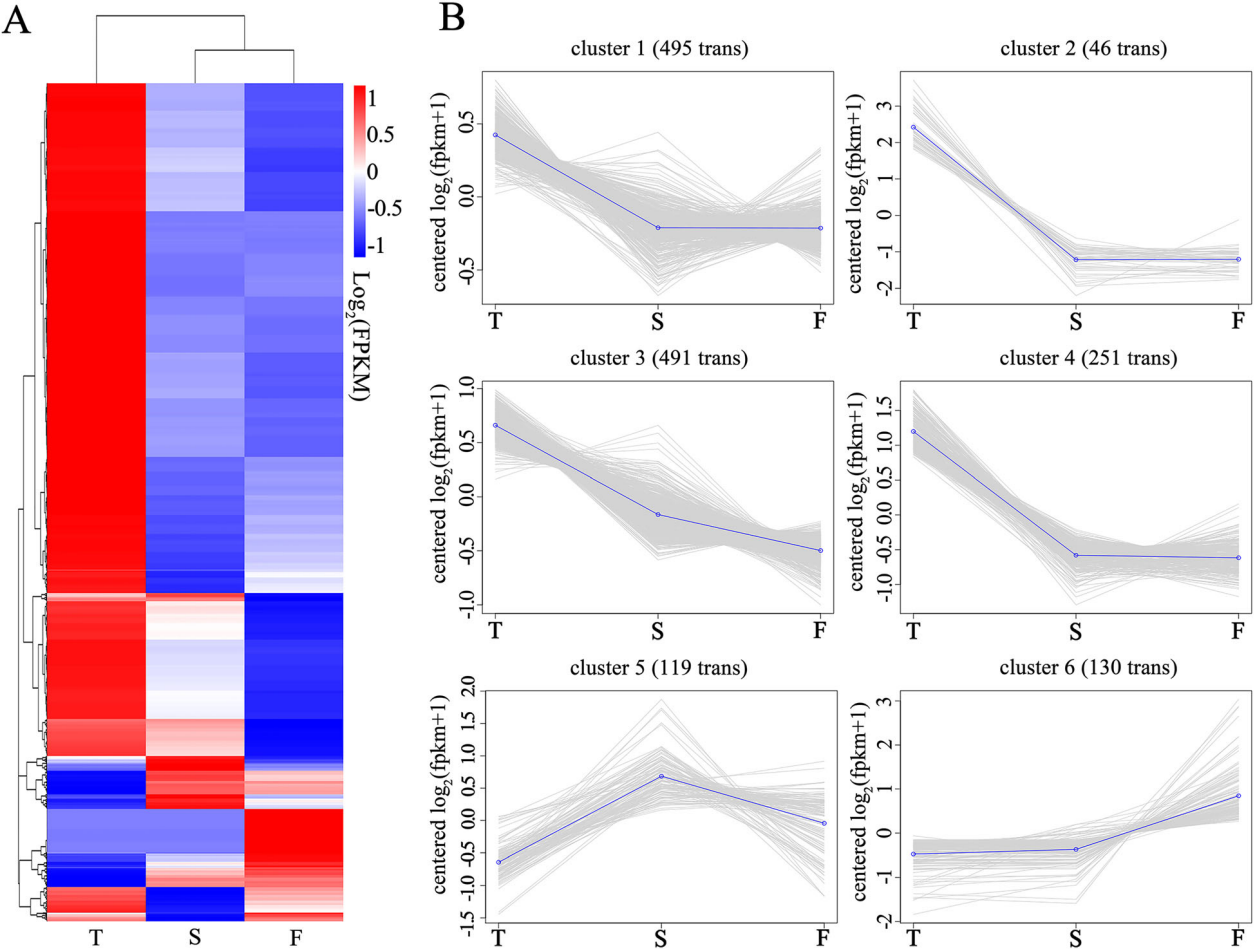
Cluster analysis of all DEGs. **a** The clustering heatmap of DEGs in T (tubular type), S (spoon type) and T (flat type). **b** The six subclusters of DEGs with different expression patterns

The overall expression pattern of 1532 DEGs are shown on the clustering heatmap (Fig. 8a), and all the DEGs were classified into six distinct expression patterns with K-means cluster analysis (Fig. 8b). Cluster 1 (495 DEGs), cluster 2 (46 DEGs), and cluster 4 (251 DEGs) had similar expression patterns, with high expression levels in the tubular type and comparable levels in the spoon and flat type. The expression pattern of cluster 6 (130 DEGs) was lower in the tubular and spoon types than the flat type, which was opposite to cluster 2. Through K-means cluster analysis, DEGs encoding TFs were concentrated in cluster 2, cluster 4, and cluster 6, and a total of 22 TFs were finally detected (Fig. 7b).

### DEGs involved in ray floret morphogenesis

To further determine the genes involved in regulating the morphology of ray florets, 45 differentially expressed TFs detected through pairwise comparison and K-means cluster analysis were filtered to obtain candidate TFs with a ten-fold or more difference in fragments per kilobase of transcript per million mapped reads (FPKM) (Additional file 8: Table S3), including six *AP2/ERFs* (*PTI5*, c55396.graph_c0; *ERF098*, c66167.graph_c0; *ERF5*, c70056.graph_c0; *ERF13*, c70872.graph_c0; *ERF1B*, c72903.graph_c0; *WRI1*, c80498.graph_c0), two *MYBs* (*WER*, c64713.graph_c0; *MYB5*, c66377.graph_c0), three *WRKY*s (*WRKY1*, c67151.graph_c0; *WRKY55*, c71617.graph_c0; *WRKY2*, c73591.graph_c1), three *bHLH* genes (*bHLH75–1*, c67890.graph_c0; *bHLH75–2*, c64947.graph_c0; *bHLH18*, c70206.graph_c0), one homolog of *NAC* (*NAC83*, c69374.graph_c0) and one *HEAT STRESS TRANSCRIPTION FACTOR* gene (*HSFB3*, c71989.graph_c1).

Furthermore, the division and expansion of plant petal cells were regulated by many genes, the expression levels of which were mostly affected by plant hormones, so further analysis of DEGs related to plant hormones was performed (Additional file 9: Table S4). The auxin response genes *SAUR* (c68047.graph_c0) and *GH3* (c68304.graph_c0 and c80649.graph_c1) were up-regulated in the tubular type compared with the spoon or flat type. In addition, *AP2/ERF* (c77988.graph_c0, c76110.graph_c0, c65396.graph_c0, c76902.graph_c0, c70056.graph_c0, c70872.graph_c0, c80498.graph_c0, c66167.graph_c0 and c72903.graph_c0) and *GA2OX1* (c66798.graph_c0) also displayed the highest expression levels in the tubular type and relatively low expression levels in the spoon and flat types, and *PYRABACTIN-*like *4* (*PYL4*, c70076.graph_c0) and *BRASSINOSTEROID INSENSITIVE1-ASSOCIATED RECEPTOR KINASE 1* (*BAK1*, c70397.graph_c0) showed the lowest expression levels in the spoon type.

### qRT-PCR validation of DEGs related to ray floret morphogenesis

**Fig. 9:**
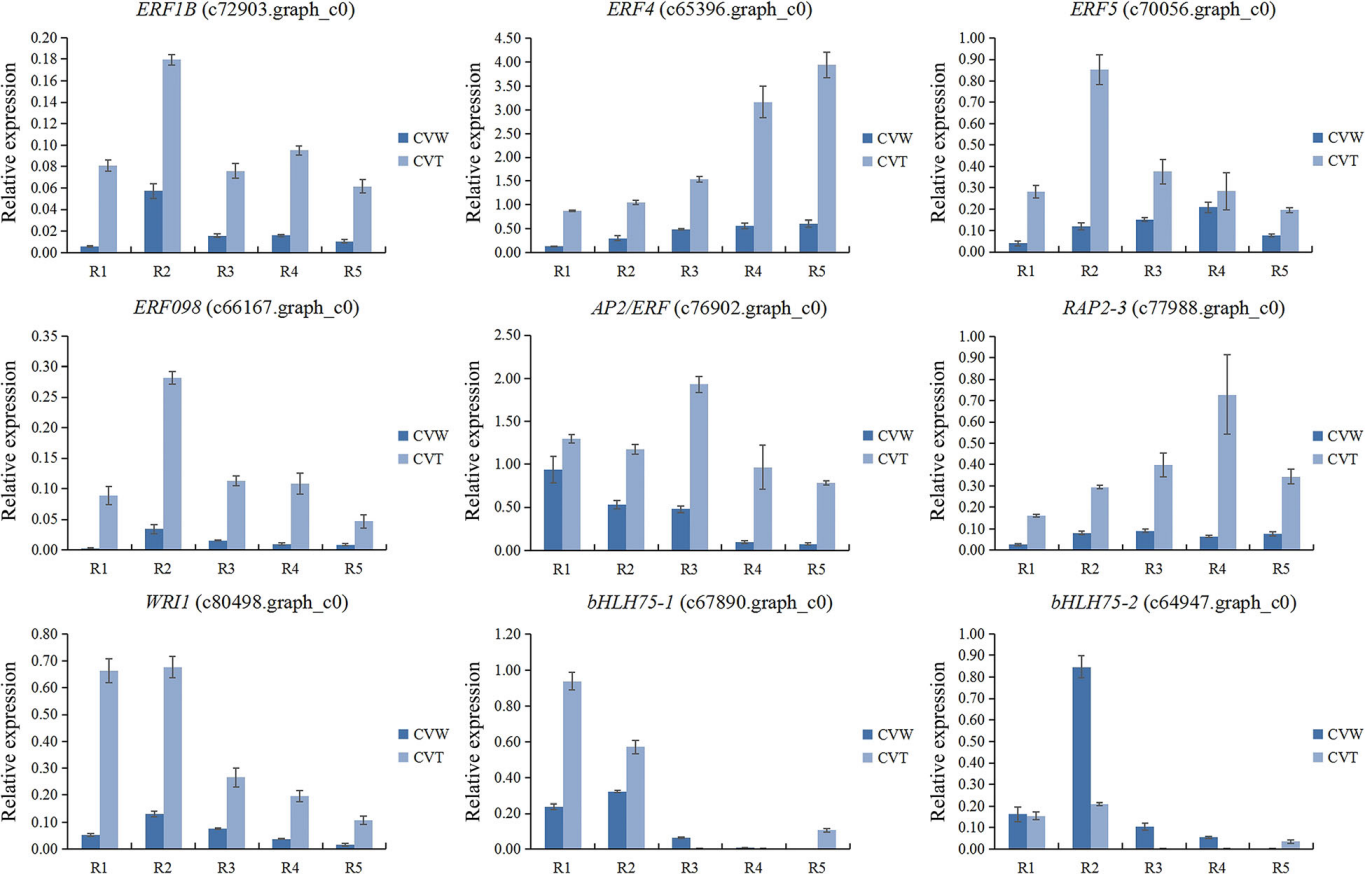
The expression patterns of DEGs encoding TFs in ray floret petals of CVW and CVT at different opening stages. R1-R5: ray florets at different opening stages

**Fig. 10:**
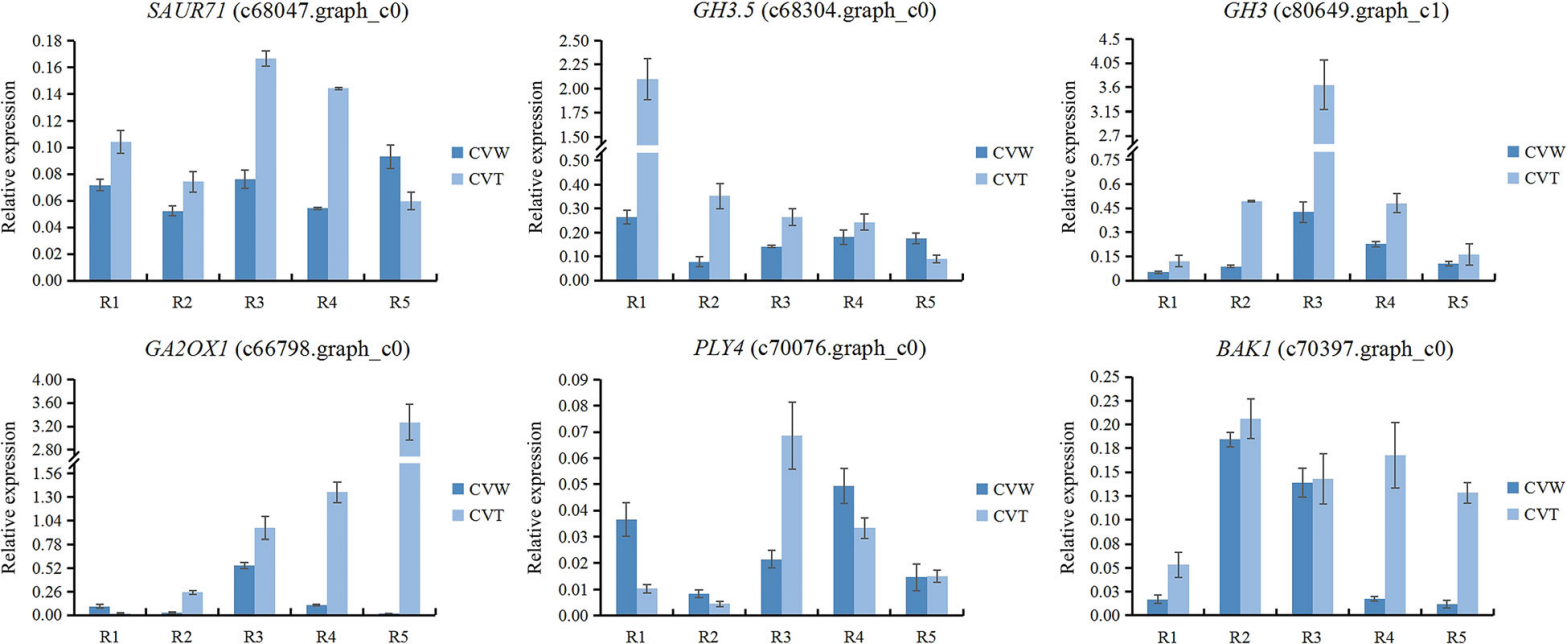
The expression patterns of DEGs related to plant hormone. R1-R5: ray florets at different opening stages

To identify key genes affecting ray floret types, 15 transcripts were selected as representatives of DEGs and analyzed in tubular, spoon and flat ray florets of CVZ (Fig. 1c) using qRT-PCR (Additional file 10: Fig. S6). To further investigate the involvement of these genes in ray floret morphogenesis, qRT-PCR was performed in ray floret petals at R1-R5 stage of CVW and CVT (Fig. 1a, b). *ERF4* (c65396.graph_c0), the expression level of which gradually increased with the gradual development of the ray florets, was expressed at high levels in CVT compared with CVW. Four genes, including *WRI1* (c80498.graph_c0), *RAP2–3* (c77988.graph_c0), *ERF098* (c66167.graph_c0), and *ERF5* (c70056.graph_c0), showed similar uniform expression patterns, which the expression levels of these genes all showed a tendency to increase and then decrease during the five opening stages and higher enrichment in CVT (Fig. 9). Moreover, *SAUR71* (c68047.graph_c0), *GH3.5* (c68304.graph_c0) and *GH3* (c80649.graph_c1) had higher expression levels in ray floret petals of CVT than CVW (Fig. 10). Additionally, *GA2OX1*(c66798.graph_c0) also showed a high expression in CVT ray floret petals. The above results further indicated that these genes were involved in the morphogenesis of ray florets and regulated the morphological differences of the tubular, spoon and flat types.

## Discussion

### Morphological analysis reveals the development of different types of ray florets

The developmental process of the flat type of ray floret in *C. vestitum* starts with the petal primordium initiation on both sides of the cup-shaped ray floret primordium in a bilaterally symmetric manner, after which the ventral petal primordia grows rapidly while the development of dorsal stagnates, and eventually, the ray floret exhibits a gap on the dorsal (Fig. 3a, c). The developmental processes of ray florets in *H. annuus* [44], *Anacyclus clavatus* [1] and *C. lavandulifolium* [45] are similar to *C. vestitum*. In addition, another type of ray floret development has been observed in *G. hybrida* [9, 44] and *S. vulgaris* [46]. Based on the cup-shaped structure, five petal primordia form and show a radiant symmetry. Subsequently, three ventral petal primordia fuse and gradually elongate and grow further, whereas the two dorsal petals remain unfused and stop growing. The morphological differences in ray florets may appear at the early stage of petal formation, that is, during the period of vigorous cell division, or in the late stage of petal growth when cell expansion occurs more than cell division [30]. Ding et al. [47] described a chrysanthemum cultivar with tubular ray florets with hooked ends, while those of its bud sport mutant were tubular with straight ends. This morphological difference in ray florets was discernible during a late stage of petal development, while in our study, morphological differences of *C. vestitum* ray florets appeared early in petal development.

### Numerous genes are involved in morphogenesis of ray florets

*MADS-box* genes determine the identity of floral organs [8, 13, 48, 49]. In the present study, the results obtained by expression analysis of some *MADS-box* (Additional file 1: Fig. S1) and the search for DEGs in the transcriptome revealed no genes with significantly different expression levels among tubular, spoon and flat ray florets. *CYC2*-like genes regulate petal symmetry of ray floret, and the expression levels of *CvCYC2b*, *CvCYC2d*, *CvCYC2e* and *CvCYC2f* were significantly higher in the tubular type than the flat type (Fig. 5). Overexpression in *S. vulgaris* of *RAY2*, a homologous gene of *CYC2e*, enables its ray florets to be transformed from the flat to the tubular type [14, 22]. Thus, we speculate that the differential expression of *CYC2*-like genes in *C. vestitum* could promote the change from the flat to the tubular type.

### Plant hormone-related DEGs regulate morphological differences in ray florets

Plant hormones are indispensable in the process of plant growth and development, and they have extremely important effects on the growth of petals. In this work, the auxin response-related genes *SAUR* (c68047.graph_c0) and *GH3* (c68304.graph_c0, c80649.graph_c1) had the highest expression levels in tubular ray floret petals of CVZ and relatively low expression levels in the spoon and flat types of CVZ (Additional file 10: Fig. S6). The expression levels in ray floret petals were also significantly higher in CVT than CVW (Fig. 10), indicating their critical role in regulating the morphological differences in ray florets. The *DR5* reporter of auxin function is expressed in the Arabidopsis floral meristem (FM) peripheral zone where floral organs will arise [50, 51], and initiation of the petal primordium depends on the activity of auxin [52]. The disruption of auxin polar transport in *pin-formed1* (*pin1*) and *pinoid* (*pid*) mutants can cause some floral organs to fail to initiate or the number and location of floral organs to be abnormal [53, 54]. Moreover, mutations of auxin biosynthesis and response-related genes can also significantly affect the number and formation of petals [28, 55]. Upon initiation of the petal primordia, auxin begins to accumulate in the developing petals and induces a series of genes regulating petal growth to function.

The morphological differences in the ray florets of *C. vestitum* appeared in the early stage of petal development (Fig. 3), when the division of petal cells was vigorous. According to statistical observations of the petal epidermal cells of ray florets with different shapes, the number of adaxial epidermal cells in tubular ray florets of CVT was significantly higher than the flat type of CVW (Fig. 4c, e). In addition, the expression levels of *AP2/ERF*, which are closely related to cell division, were much higher in the tubular type of CVT than the flat type of CVW (Fig. 9). It has been reported that *AUXIN REGULATED GENE INVOLVED IN ORGAN GROWTH* (*ARGOS*) acts upstream of *AINTEGUMENTA* (*ANT*) [31, 32], which is a subfamily member of *AP2/ERF*. These subfamilies have similar functions in promoting cell division [34], some of which are involved in regulating petal cell proliferation [35]. In addition, the auxin response genes *MONOPTEROS* (*MP*)/*ARF5* activate *AINTEGUMENTA-LIKE 6* (*AIL6*) [33], which is closely related to and functions redundantly with *ANT* to regulate petal development [56]. It is speculated that at the early stage of petal formation, petal cell division of tubular ray florets is more vigorous than the flat type, which is regulated by *AP2/ERF* and results in the formation of ring shape petal primordia and the tubular type. However, because of the low expression of *AP2/ERF* in the flat type, petal cell division ability is weak, and growth of dorsal petal primordia is stagnant, eventually forming the flat type.

## Conclusion

**Fig. 11:**
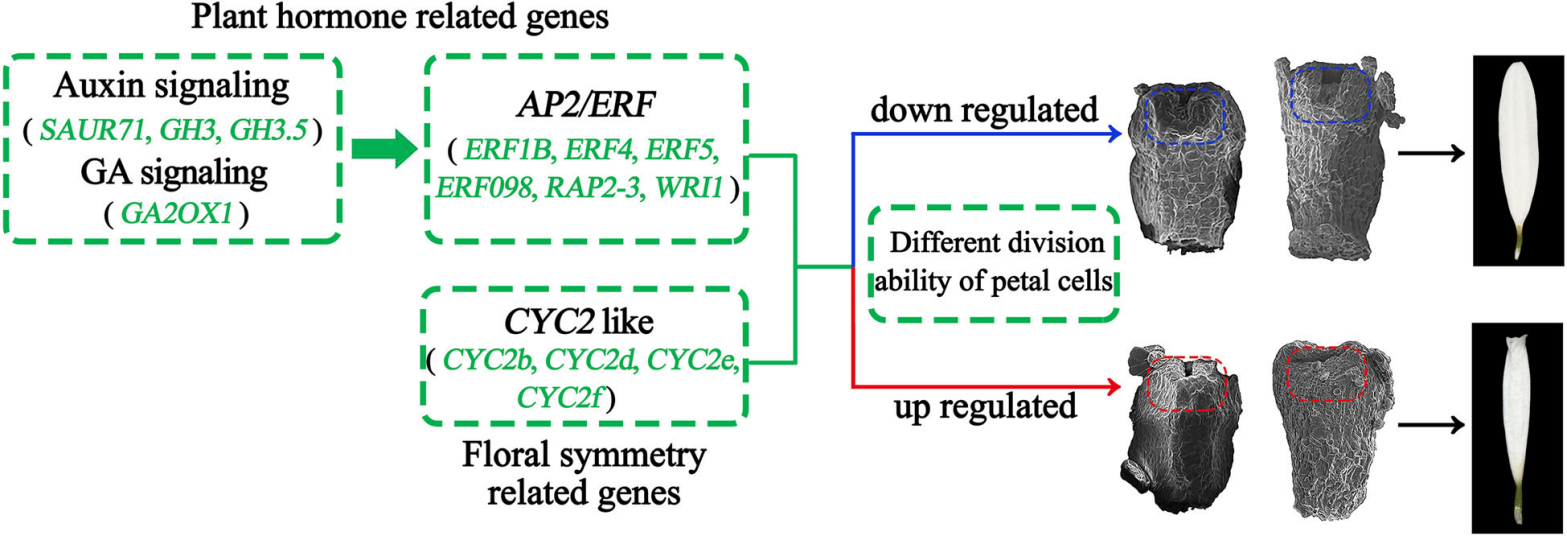
Summary of plant hormone-related and floral symmetry-related gene regulation of different morphological ray florets

Based on morphological observation and transcriptomic analysis combined with gene expression analysis, we found that morphological differences appeared in the early stage of ray floret development, the division of petal cells was more vigorous in tubular ray floret than flat type and the expression levels of *CvCYC2b*, *CvCYC2d*, *CvCYC2e*, and *CvCYC2f* involved in floral symmetry and *CvAP2/ERF*, *CvSAUR71*, *CvGH3*, *CvGH3.5*, and *CvGA2OX1* involved in plant hormones were higher in tubular ray floret than flat type. Based on the above findings and previous studies, the mechanism underlying ray floret morphogenesis is summarized in Fig. 11. We speculated that up-regulated expression of auxin and gibberellin-related genes in tubular ray florets might promote the increase in downstream *AP2/ERF* gene expression, thereby enhancing cell division ability and promoting the petal primordium to form an intact ring shape and eventually develop into a tubular type. Concurrently, *CYC2*-like genes are involved in common regulation. Overall, this study provides direction in identifying the mechanism underlying the development of different morphological ray florets and enriches our understanding of ray floret morphogenesis in chrysanthemum.

## Methods

### Plant materials and growth conditions

*C. vestitum* distributed in the central China with a large population is a major origin hexaploid species (2n = 6x = 54) of cultivated chrysanthemum [41] and can be collected non-destructively through cuttings for ex-situ conservation. The cuttings of *C. vestitum* with different types of ray florets were collected from their native habitat area and transplanted to the greenhouse [41, 43]. All experiments were adhered to Regulations of the People’s Republic of China for Wild Plants Protection.

Three plant lines of *C. vestitum*, CVW, CVT and CVZ, were grown in the chrysanthemum germplasm nursery of the Beijing Forestry University, Beijing, China. Among these plant lines, the ray florets of CVW are all flat type (Fig. 1a), those of CVT are all tubular type (Fig. 1b), and CVZ has three ray floret shapes of the flat, spoon and tubular type (Fig. 1c). CVW and CVT were used for morphological observation and gene expression analysis. The ray floret petals of CVZ were selected for RNA-Seq analysis, as the flat, spoon and tubular types were on the same capitulum and had the same genetic background, which is more conducive to explore genes that effectively regulate the difference in ray florets.

### Light microscopy observations

The apical buds of CVW and CVT from the vegetative growth period to reproductive growth period were fixed in FAA (50% ethanol, 38% formaldehyde solution, glacial acetic acid = 18, 1, 1). Then, the specimens were dehydrated through an ethanol series (50, 70, 85, 95, 100%) and transferred to a xylene-ethanol series up to 100% xylene. The specific method used has been described by Wen et al. [45].

### SEM observations

The apical buds of CVW and CVT from the vegetative growth period to reproductive growth period were collected and placed in 2.5% glutaraldehyde fixative for at least 12 h. An ethanol series (30, 50, 70, 90, 95, 100%) was used for material dehydration, followed by an ethanol-tert-butanol series up to 100% tert-butanol. The samples were freeze-dried overnight using a lyophilizer (ES-2030; Tokyo; HITACHI; Japan) and then dissected and attached onto carbon conductive tabs. The materials were coated with an ion sputtering apparatus (E-1010; Tokyo; HITACHI; Japan) and observed by SEM (S-3400 N II; Tokyo; HITACHI; Japan).

Ray florets at the R1-R5 stage of CVW and CVT (Fig. 1a2, b2) were treated as described above. The center of the basal, middle and top regions of ray floret petals were sampled for morphological characterization of the adaxial and abaxial epidermal cells under a field of view magnified 800 times with SEM. The cell number measurement was performed using ImageJ software (http://rsb.info.nih.gov/ij/, NIH, MD, USA).

### RNA-Seq, functional annotation and data processing

The flat, spoon and tubular ray floret petals at R5 stage of CVZ (Fig. 1 c2) were sampled to construct nine libraries (F1, F2, F3, S1, S2, S3, T1, T2, T3) for RNA-Seq. Total RNAs were extracted using a Plant RNA Rapid Extraction Kit (HUAYUEYANG Biotechnology, Beijing, China) and treated with RNase-free DNaseI to digest DNA. After assessing the purity and integrity of the RNA with the Agilent 2100 Bioanalyzer and the ABI StepOnePlus Real-Time PCR System, the constructed libraries were sequenced on an Illumina HiSeq 2500 sequencing platform (Illumina, San Diego, CA, USA) by Biomarker Technologies Corporation (Beijing, China). Connectors of the raw reads and low-quality sequences were removed to obtain clean reads, which were then assembled using Trinity [57]. The obtained unigene sequences were aligned using BLAST (E-value < 10^− 5^) to the NR, Swiss-Prot, GO, COG, KOG, eggNOG, and KEGG databases and then annotated.

The reads obtained from sequencing were compared with the unigene library using Bowtie [58]. Based on the comparison results combined with RSEM [59], the transcript abundance was estimated. The expression level of unigenes was measured by FPKM [60], and differential expression among F, S and T was analyzed with DESeq2 [61]. DEGs were screened by a false discovery rate (FDR) < 0.01 and fold change (FC) ≥ 2. In addition, key DEGs were obtained using Venn and cluster analyses.

### RT-PCR analysis

The total RNAs of ray floret petals at the R1-R5 stages of CVW and CVT (Fig. 1a2, b2) and ray floret petals and disc floret corolla tubes of CVZ (Fig. 1c2) were extracted using a Plant RNA Rapid Extraction Kit (HUAYUEYANG Biotechnology, Beijing, China) and were used to synthesize cDNA for RT-PCR with the transcription kit. According to the specific procedure described by Huang et al. [23] using *Actin* as a reference gene, the genes closely related to petal morphogenesis, including *MADS-box*, *TCP*, *NAC*, *ARF*, and *WOX*, were analyzed. The primer sequences are shown in Additional file 11: Table S5.

### qRT-PCR validation

To verify the accuracy of the transcriptome data of genes related to ray floret morphogenesis, qRT-PCR, prepared using the SYBR Premix Ex Taq kit (Takara, Japan), was performed on a CFX96™ real-time system (Bio-Rad Laboratories, Hercules, CA, USA) using the procedure described by Huang et al. [23]. The primer information is listed in Additional file 12: Table S6. The relative gene expression was normalized by comparison to the expression of *SAND* in *C. vestitum*, and the analysis was performed using the 2^-ΔΔCT^ method [62]. The data are presented as the mean ± SD.

## Supplementary information

**Additional file 1: Figure S1.** Expression analysis of flower development related genes in different ray floret petals of CVW CVT and CVZ using RT-PCR. The expression level of actin is used to normalize the mRNA levels for each sample. R1-R5 indicated the five opening stages of ray floret petals, F: flat ray floret petal, S: spoon ray floret petal, T: tubular ray floret petal, D: disc floret corolla tube.

**Additional file 2: Figure S2.** The original and full-length gel images of flower development related genes. R1-R5 indicated the five opening stages of ray floret petals, F: flat ray floret petal, S: spoon ray floret petal, T: tubular ray floret petal, D: disc floret corolla tube.

**Additional file 3: Figure S3.** PCA analysis of the 9 samples (T1, T2, T3, S1, S2, S3, F1, F2, F3). Each group contains three biological replications.

**Additional file 4: Table S1.** Summary statistics of clean reads in the transcriptomes of *Chrysanthemum vestitum*.

**Additional file 5: Figure S4.** All genes annotation in public databases.

**Additional file 6: Table S2.** Differential expression information and annotation of DEGs in the Venn analysis.

**Additional file 7: Figure S5.** GO terms (A-C) and KEGG pathways (D-F) significantly enriched in DEGs in comparisons of T (tubular ray floret petal), S (spoon ray floret petal) and F (flat ray floret petal).

**Additional file 8 Table S3.** The DEGs encoding transcription factors with a ten-fold or more difference in FPKM.

**Additional file 9 Table S4.** The DEGs related to plant hormones.

**Additional file 10: Figure S6.** qRT-PCR analysis of 15 DEGs in T (tubular ray floret petal), S (spoon ray floret petal) and F (flat ray floret petal) of CVZ.

**Additional file 11: Table S5.** Primer sequences used in RT-PCR experiments.

**Additional file 12: Table S6.** Primer sequences used in qRT-PCR experiments.

## Data Availability

The datasets supporting the results presented in this study are included in this article (and its additional files). The raw data for the 9 sequenced libraries are available in the NCBI SRA database with accession number PRJNA637210.

## Abbreviations

ABA: Abscisic acid
AIL6: AINTEGUMENTA-LIKE 6
ANT: AINTEGUMENTA
AP2/ERF: APETALA2/ETHYLENE RESPONSIVE FACTOR
ARF: AUXIN RESPONSE FACTOR
ARGOS: AUXIN REGULATED GENE INVOLVED IN ORGAN GROWTH
BAK1: BRASSINOSTEROID INSENSITIVE1-ASSOCIATED RECEPTOR KINASE 1
bHLH: Basic helix-loop-helix
BR: Brassinolide
CTK: Cytokinin
CTMD: The corolla tube merged degree
CYC: CYCLOIDEA
DEGs: Differentially expressed genes
DFP: Disc floret primordia
DICH: DICHOTOMA
FC: Fold change
FDR: False discovery rate
FM: Floral meristem
FPKM: Fragments per kilobase of transcript per million mapped reads
GA: Gibberellin
GA2OX1: GIBBERELLIN 2-BETA-DIOXYGENASE 1
GDEF: GERBERA DEFICIENS-LIKE
GGLO1: GERBERA GLOBOSA-LIKE1
GH3: GRETCHEN HAGEN3
GRCD: GERBERA REGULATOR of CAPITULUM DEVELOPMENT
HSFB3: HEAT STRESS TRANSCRIPTION FACTOR 3
MADS: MCM1, AG, DEFA and SRF
MP: MONOPTEROS
NAC: NAM/ATAF/CUC
pid: Pinoid
pin1: Pin-formed1
PYL4: PYRABACTIN-like 4
qRT-PCR: Real-time quantitative polymerase chain reaction
QTL: Quantitative trait locus
RFP: Ray floret primordia
RNRF: The relative number of ray florets
RT-PCR: Semi-quantitative reverse transcriptase-polymerase chain reaction
SAUR: SMALL AUXIN UPREGULATED RNA
SEM: Scanning electron microscopy
TCP: TEOSINTE BRANCHED/CYCLOIDEA/PCF
TF: Transcription factor
WOX: WUSCHEL-like homeobox
