# Supplementary figures and images for: Comprehensive transcriptomic analysis provides new insights into the mechanism of ray floret morphogenesis in chrysanthemum
Source: BMC Genomics. 2020 Oct 20;21:728. doi: 10.1186/s12864-020-07110-y (PMC7574349; doi:10.1186/s12864-020-07110-y)

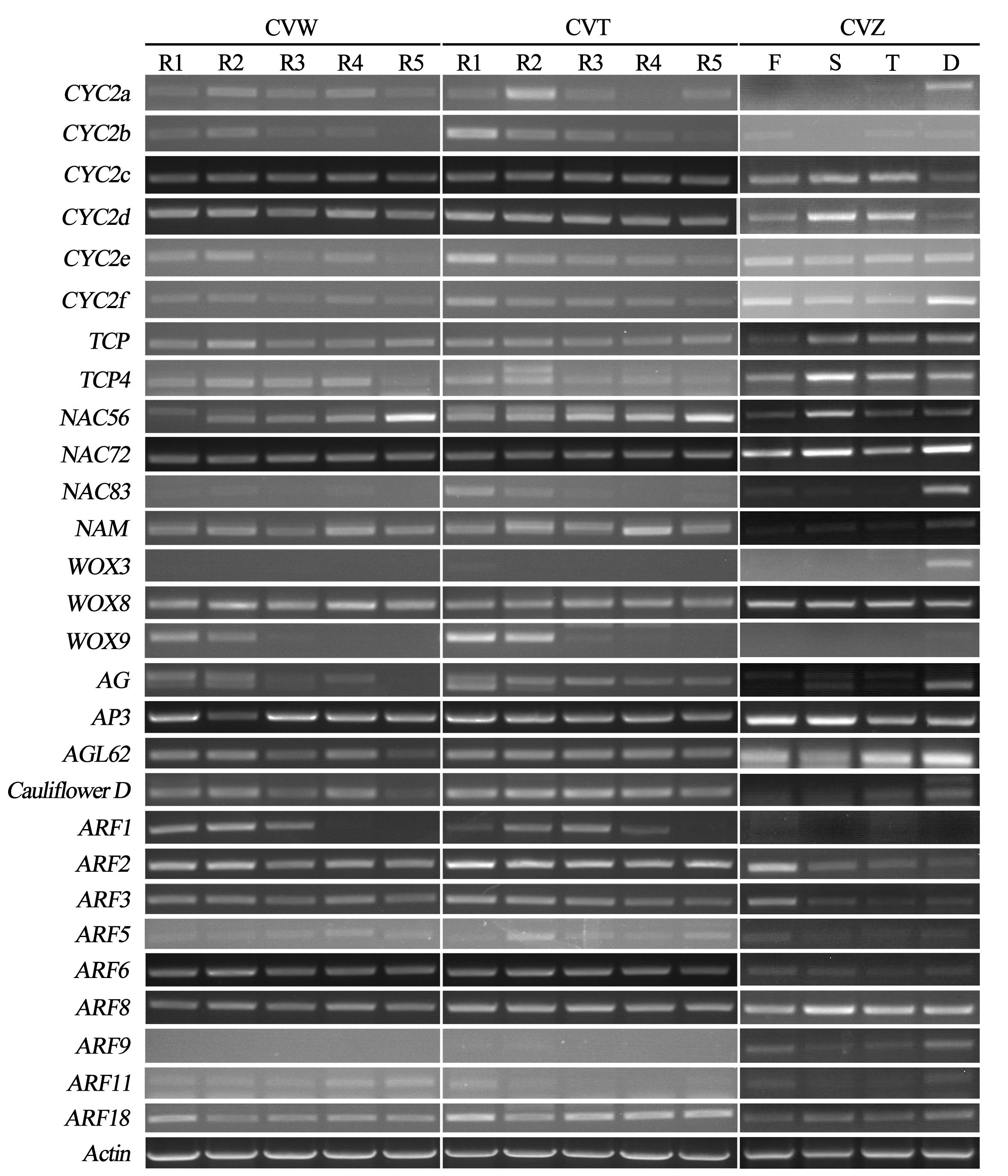

Supplement: Supplementary file 1 — Additional file 1: Figure S1. Expression analysis of flower development related genes in different ray floret petals of CVW CVT and CVZ using RT-PCR. The expression level of actin is used to normalize the mRNA levels for each sample. R1-R5 indicated the five opening stages of ray floret petals, F: flat ray floret petal, S: spoon ray floret petal, T: tubular ray floret petal, D: disc floret corolla tube. [file 12864_2020_7110_MOESM1_ESM.jpg]

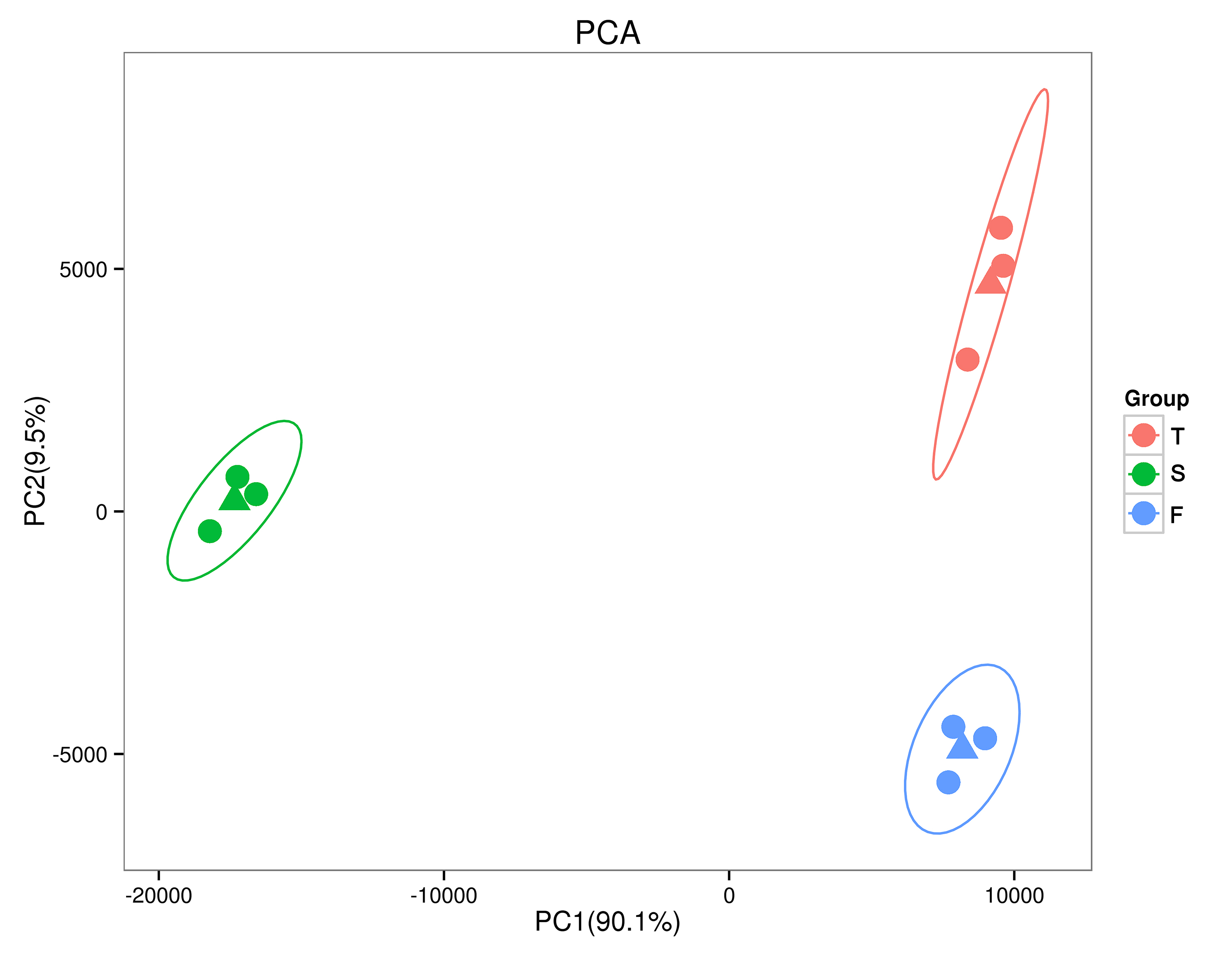

Supplement: Supplementary file 3 — Additional file 3: Figure S3. PCA analysis of the 9 samples (T1, T2, T3, S1, S2, S3, F1, F2, F3). Each group contains three biological replications. [file 12864_2020_7110_MOESM3_ESM.jpg]

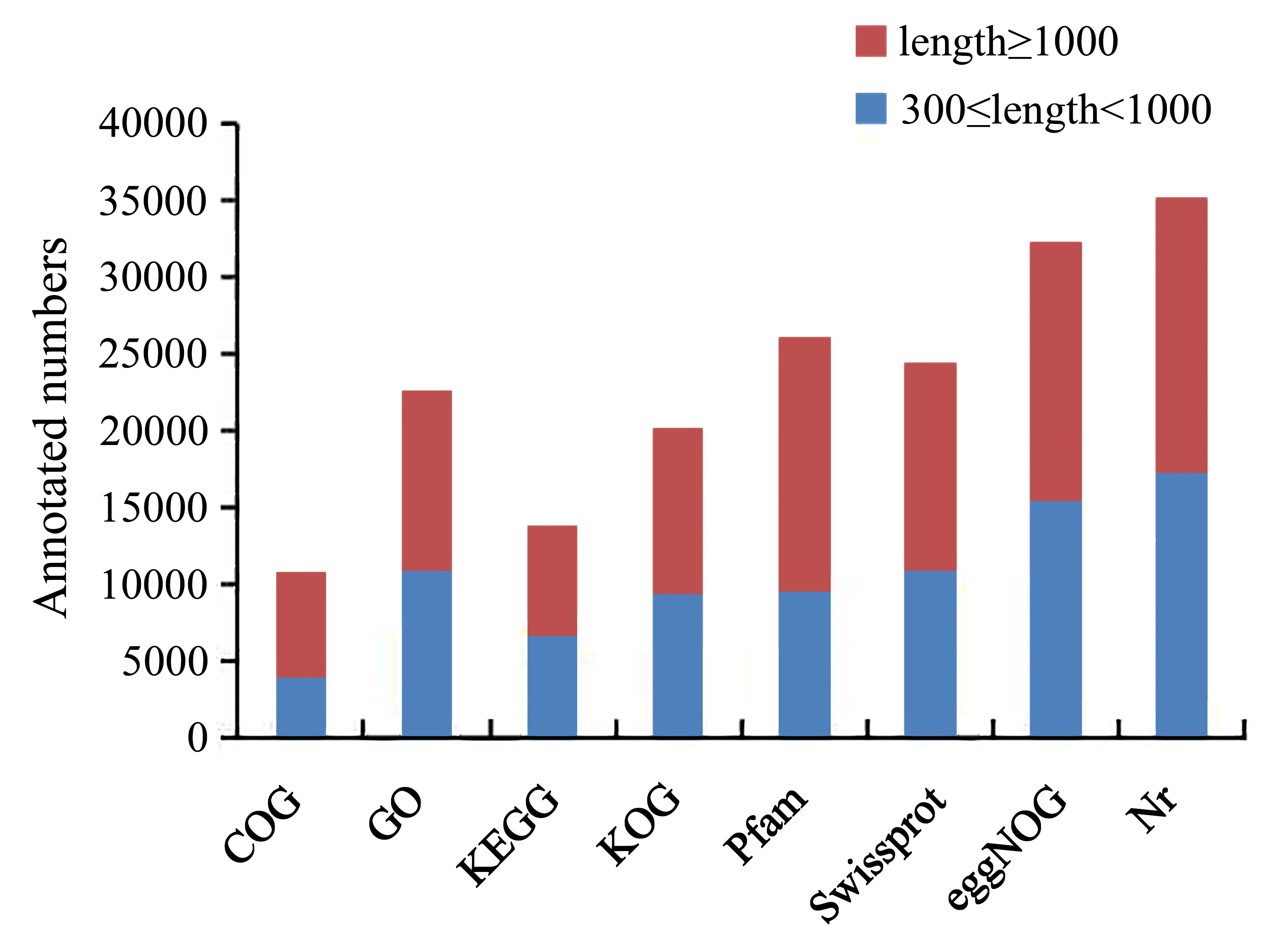

Supplement: Supplementary file 5 — Additional file 5: Figure S4. All genes annotation in public databases. [file 12864_2020_7110_MOESM5_ESM.jpg]

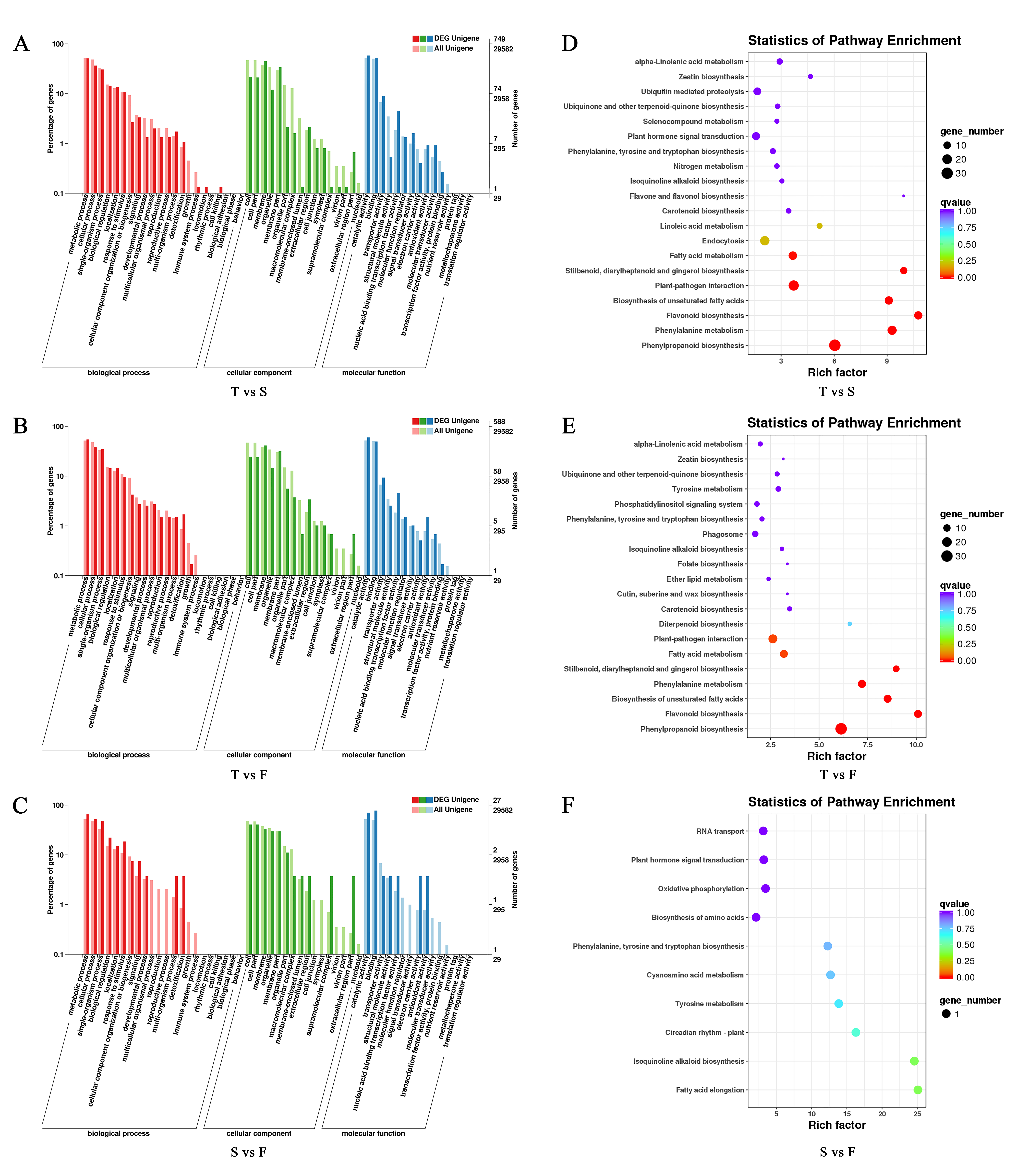

Supplement: Supplementary file 7 — Additional file 7: Figure S5. GO terms (A-C) and KEGG pathways (D-F) significantly enriched in DEGs in comparisons of T (tubular ray floret petal), S (spoon ray floret petal) and F (flat ray floret petal). [file 12864_2020_7110_MOESM7_ESM.jpg]

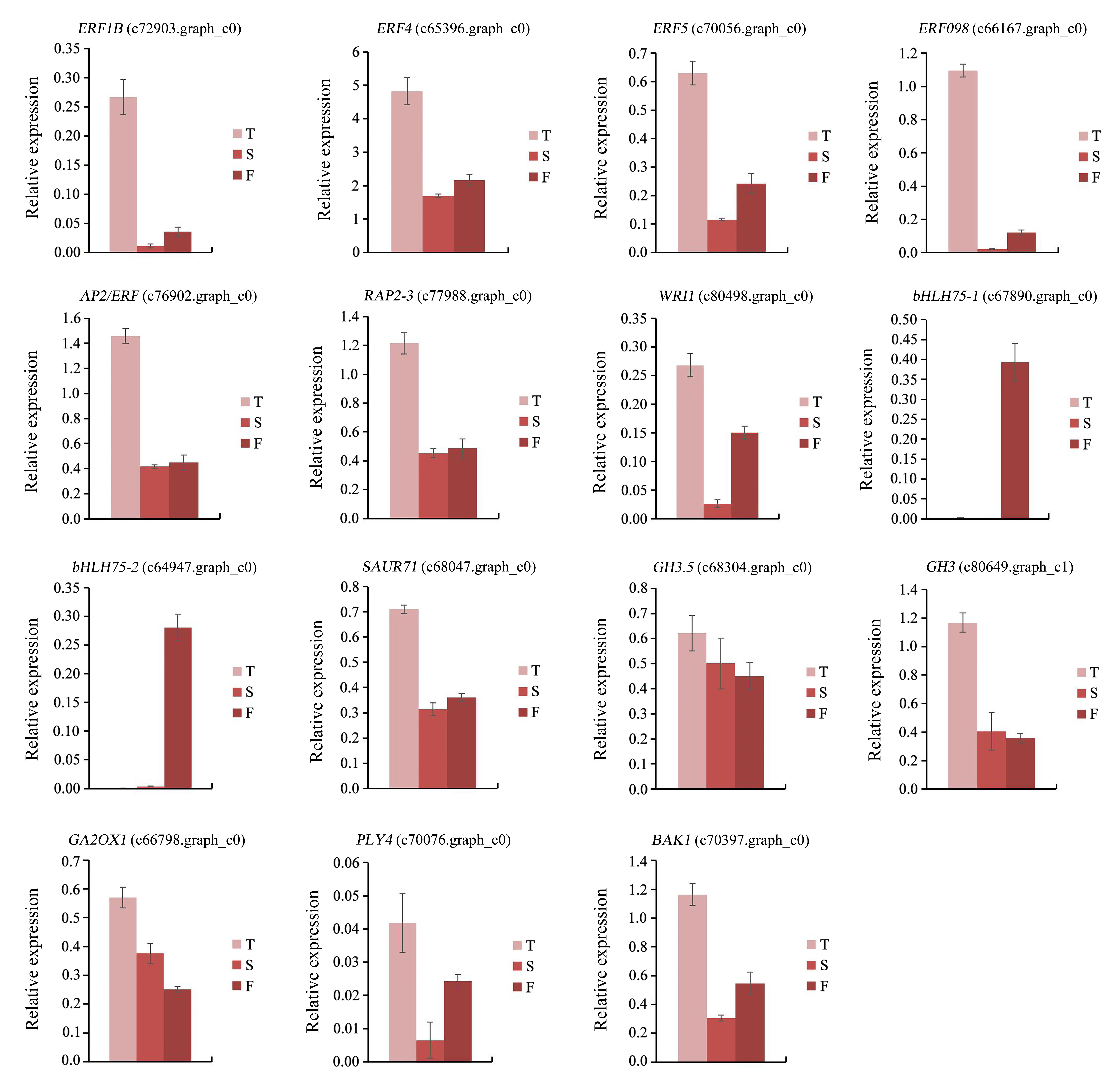

Supplement: Supplementary file 10 — Additional file 10: Figure S6. qRT-PCR analysis of 15 DEGs in T (tubular ray floret petal), S (spoon ray floret petal) and F (flat ray floret petal) of CVZ. [file 12864_2020_7110_MOESM10_ESM.jpg]
