# Supplementary material for: Comprehensive transcriptomic analysis provides new insights into the mechanism of ray floret morphogenesis in chrysanthemum
Source: BMC Genomics. 2020 Oct 20;21:728. doi: 10.1186/s12864-020-07110-y (PMC7574349; doi:10.1186/s12864-020-07110-y)
Supplement: Supplementary file 2 — Additional file 2: Figure S2. The original and full-length gel images of flower development related genes. R1-R5 indicated the five opening stages of ray floret petals, F: flat ray floret petal, S: spoon ray floret petal, T: tubular ray floret petal, D: disc floret corolla tube. [file 12864_2020_7110_MOESM2_ESM.docx]

**Additional file 2: Figure S2.** The original and full-length gel images of flower development related genes. R1-R5 indicated the five opening stages of ray floret petals, F: flat ray floret petal, S: spoon ray floret petal, T: tubular ray floret petal, D: disc floret corolla tube.


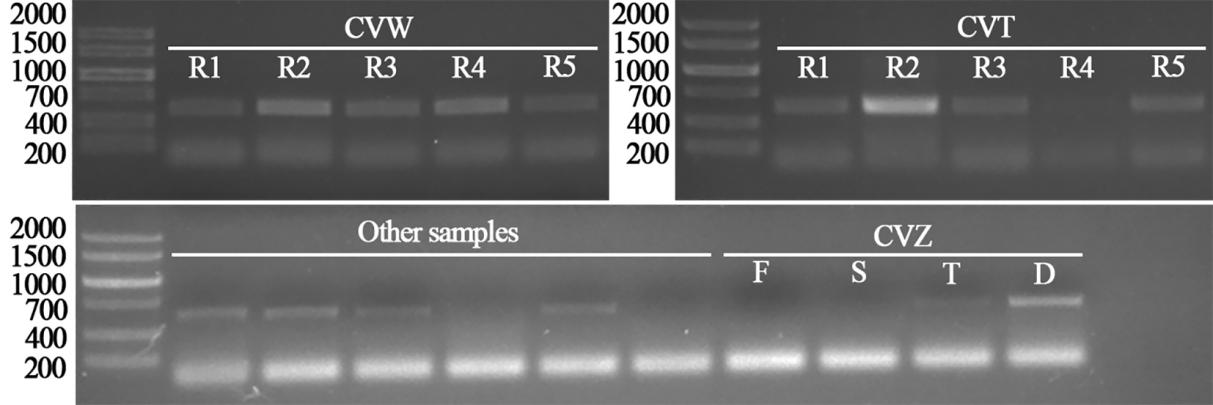


Figure S2-1. Expression analysis of *CYC2a*. Other samples refer to the materials unrelated to this study.


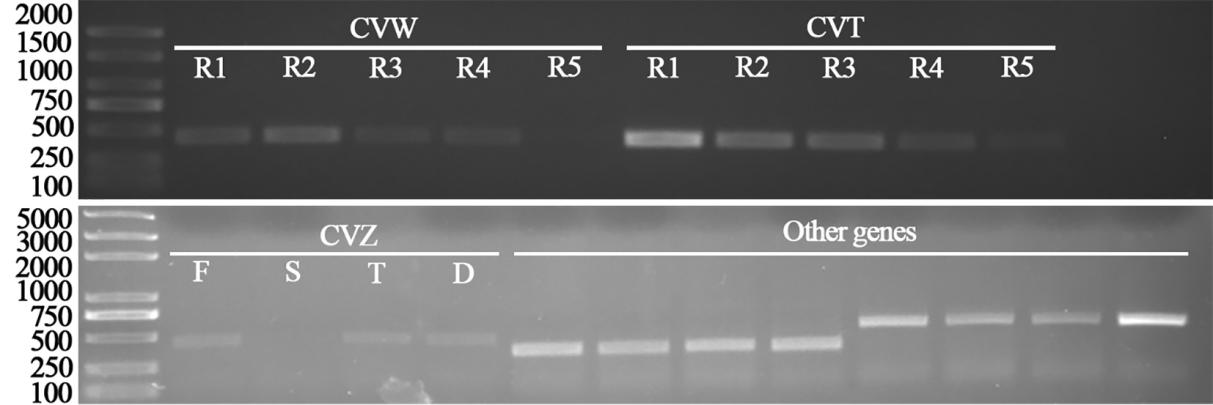


Figure S2-2. Expression analysis of *CYC2b*.


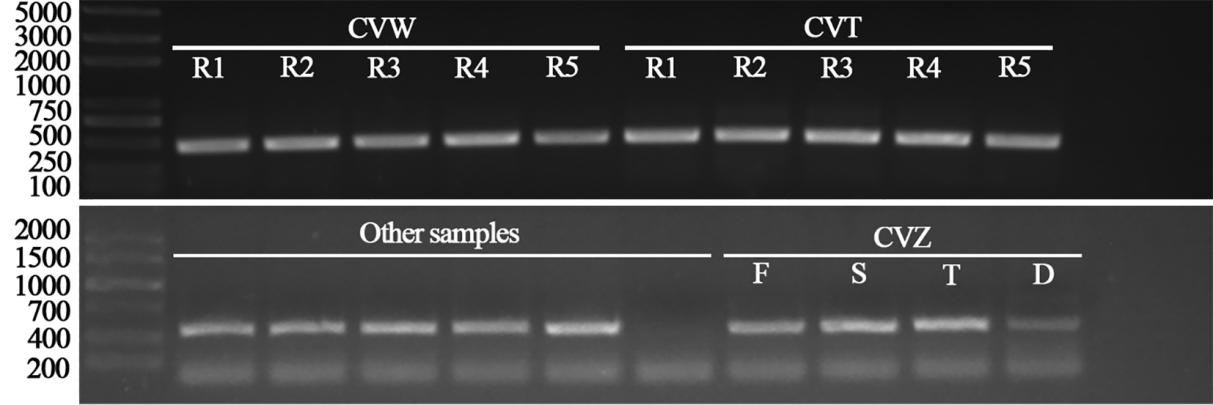


Figure S2-3. Expression analysis of *CYC2c*. Other samples refer to the materials unrelated to this study.


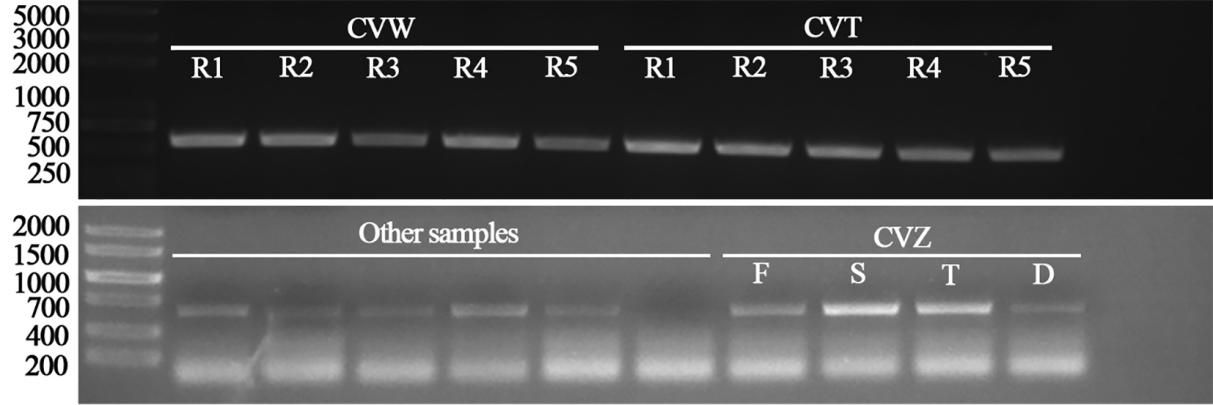


Figure S2-4. Expression analysis of *CYC2d*. Other samples refer to the materials unrelated to this study.


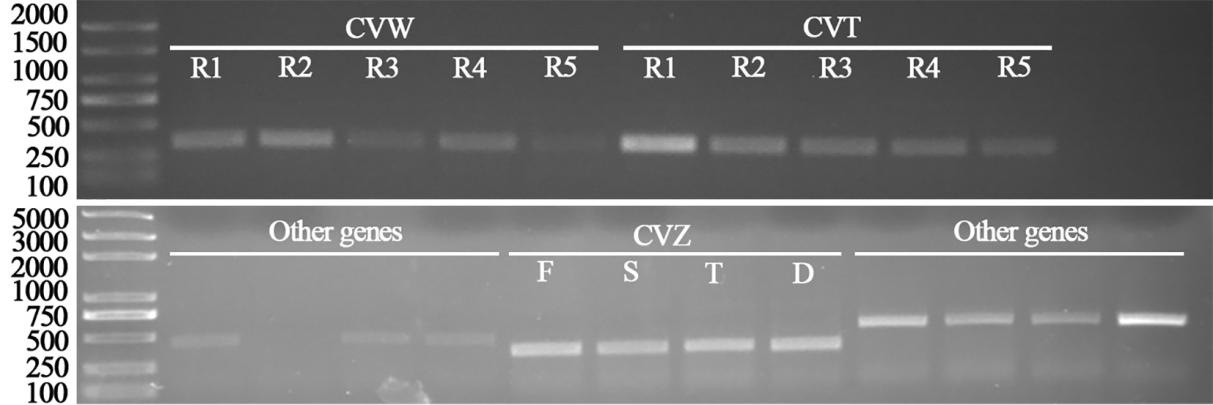


Figure S2-5. Expression analysis of *CYC2e*.


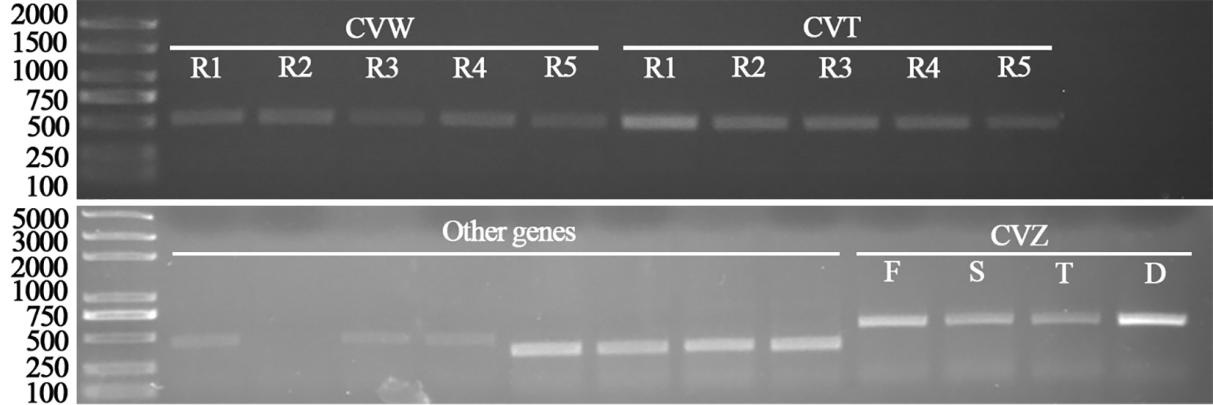


Figure S2-6. Expression analysis of *CYC2f*.


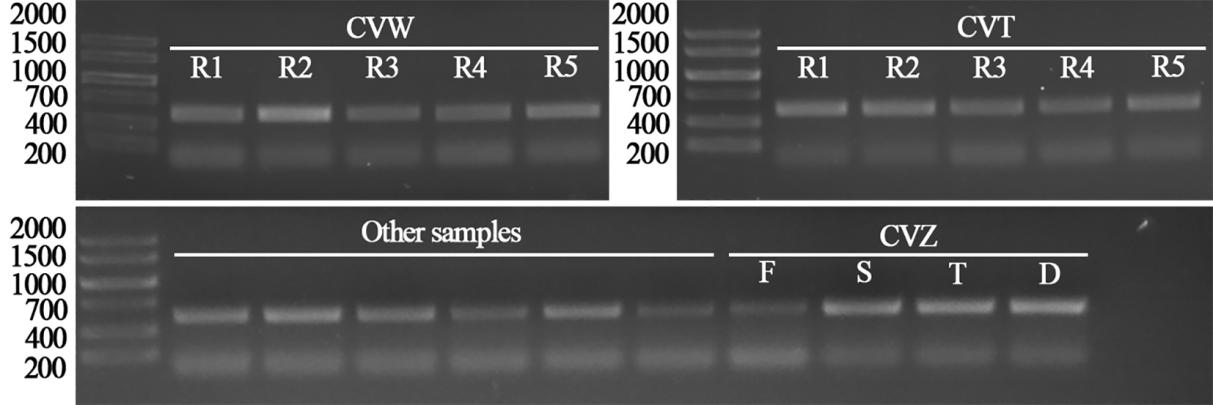


Figure S2-7. Expression analysis of *TCP*. Other samples refer to the materials unrelated to this study.


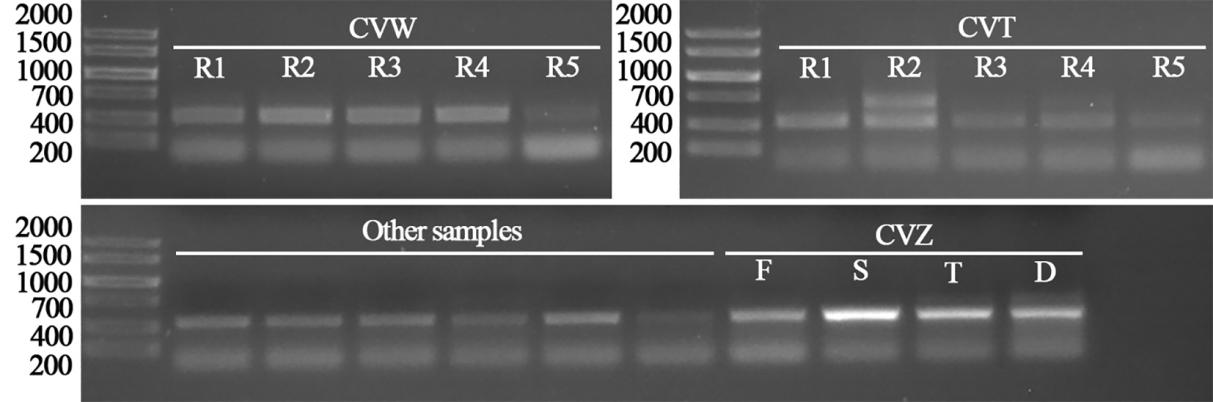


Figure S2-8. Expression analysis of *TCP4*. Other samples refer to the materials unrelated to this study.


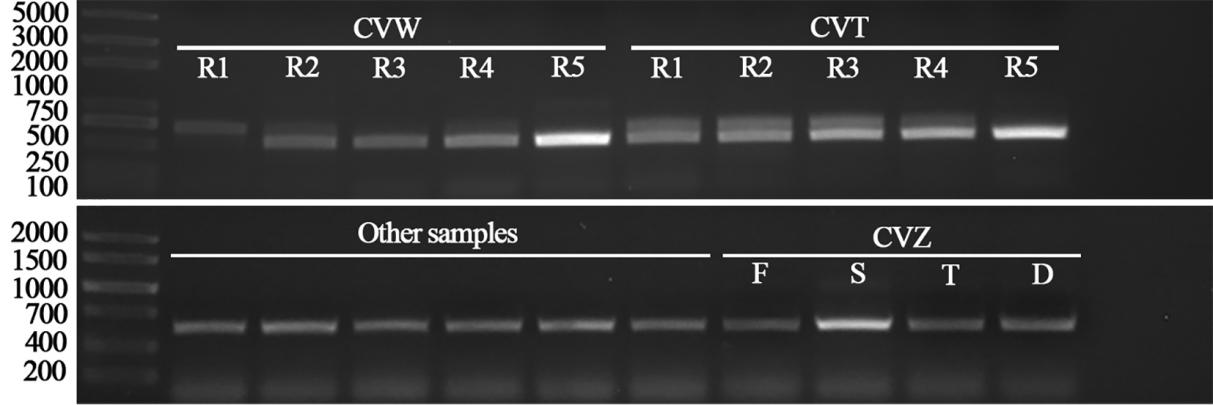


Figure S2-9. Expression analysis of *NAC56*. Other samples refer to the materials unrelated to this study.


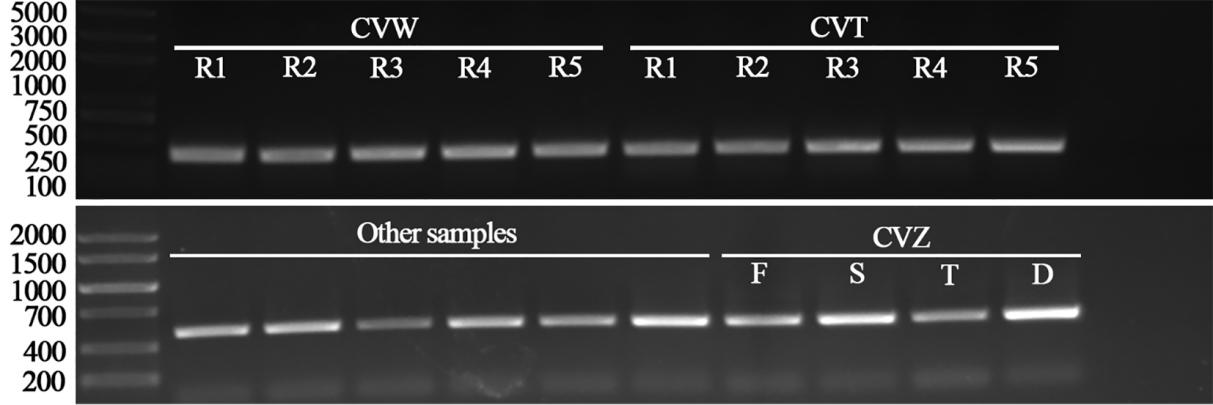


Figure S2-10. Expression analysis of *NAC72*. Other samples refer to the materials unrelated to this study.


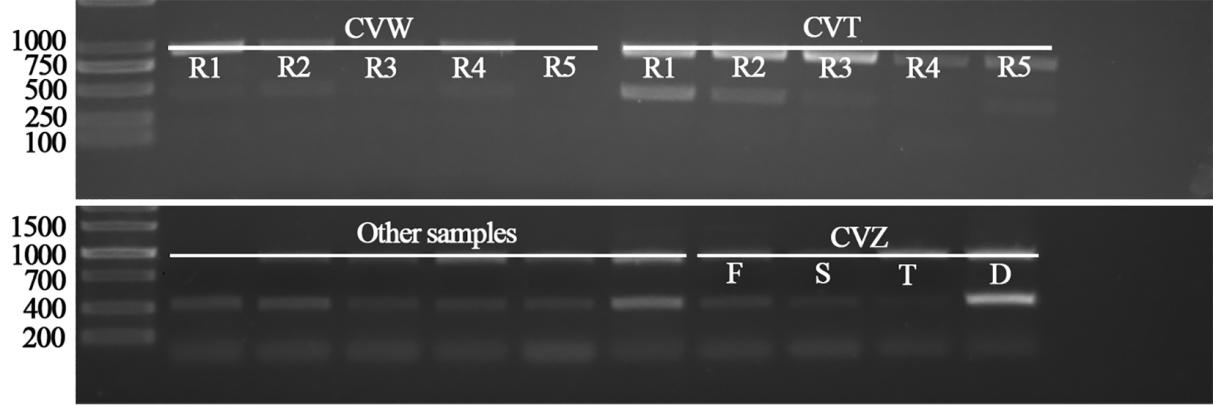


Figure S2-11. Expression analysis of *NAC83*. Other samples refer to the materials unrelated to this study.


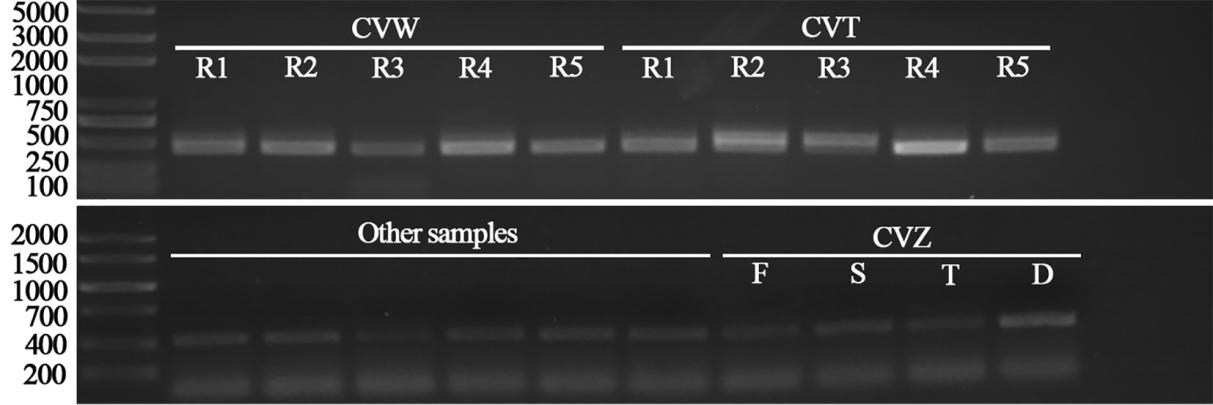


Figure S2-12. Expression analysis of *NAM*. Other samples refer to the materials unrelated to this study.


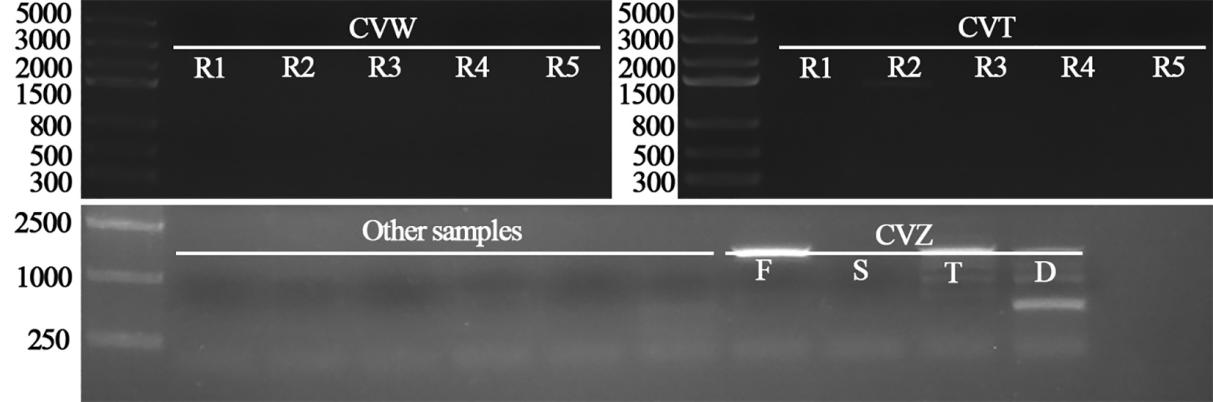


Figure S2-13. Expression analysis of *WOX3*. Other samples refer to the materials unrelated to this study.


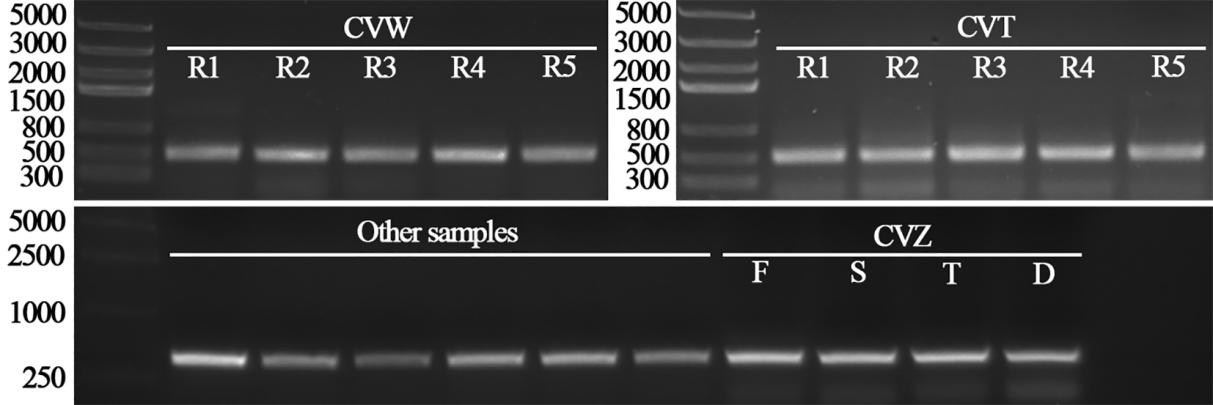


Figure S2-14. Expression analysis of *WOX8*. Other samples refer to the materials unrelated to this study.


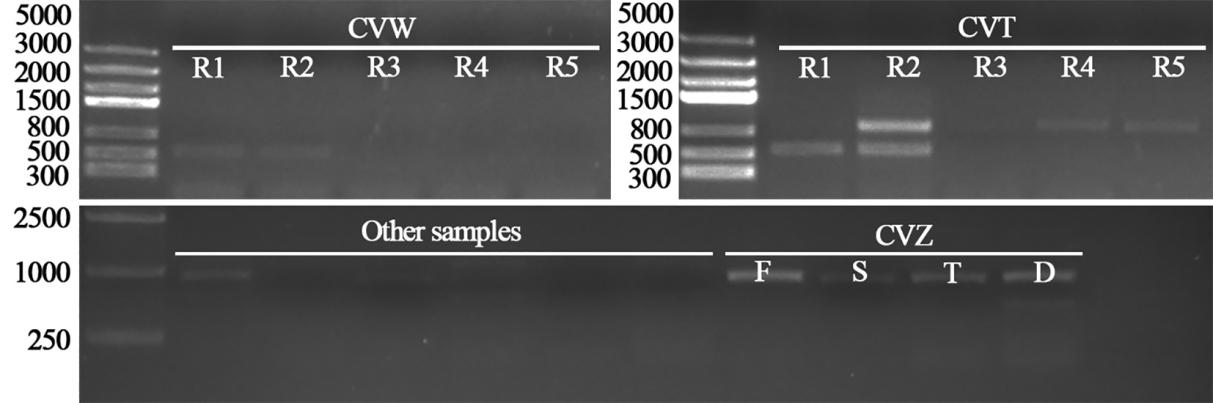


Figure S2-15. Expression analysis of *WOX9*. Other samples refer to the materials unrelated to this study.


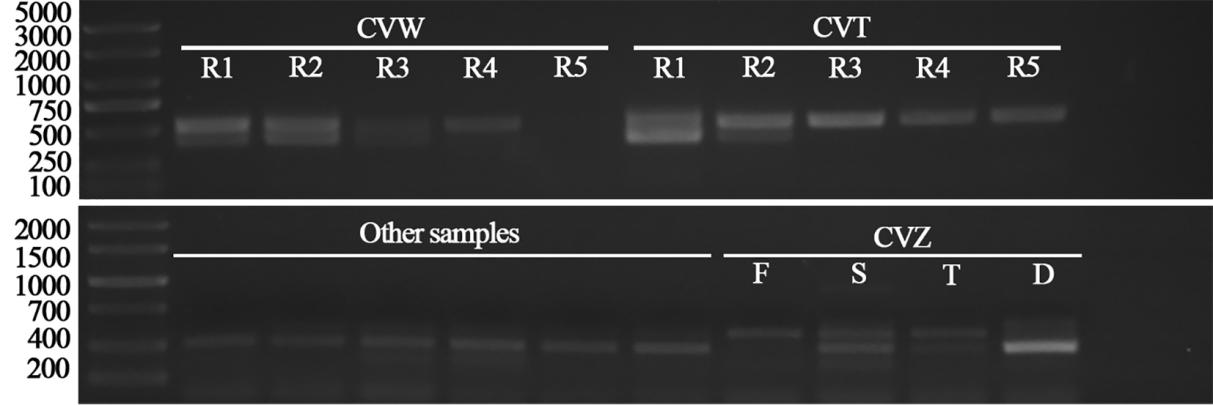


Figure S2-16. Expression analysis of *AG*. Other samples refer to the materials unrelated to this study.


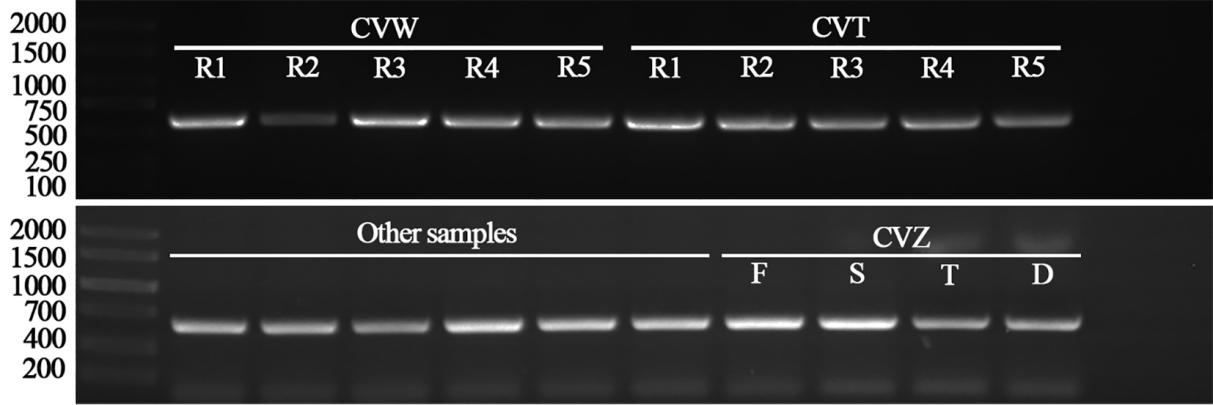


Figure S2-17. Expression analysis of *AP3*. Other samples refer to the materials unrelated to this study.


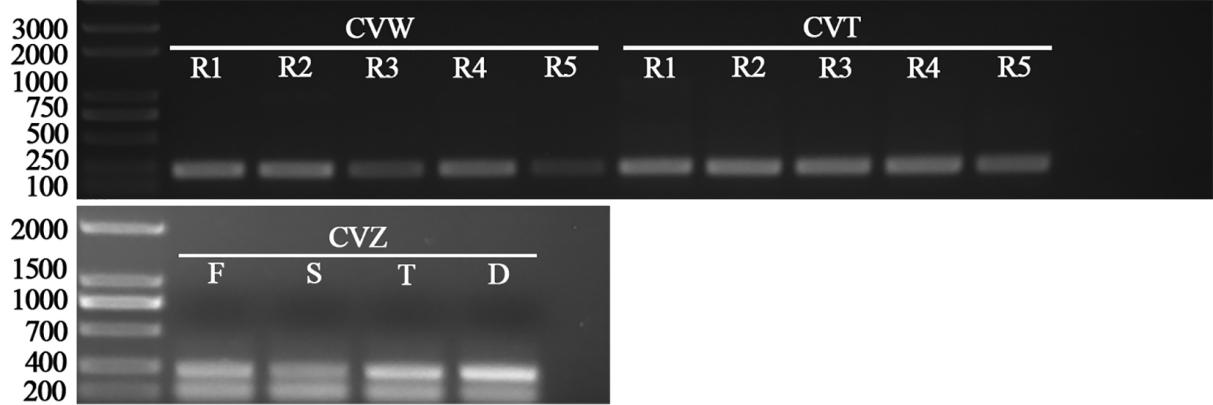


Figure S2-18. Expression analysis of *AGL62*. Other samples refer to the materials unrelated to this study.


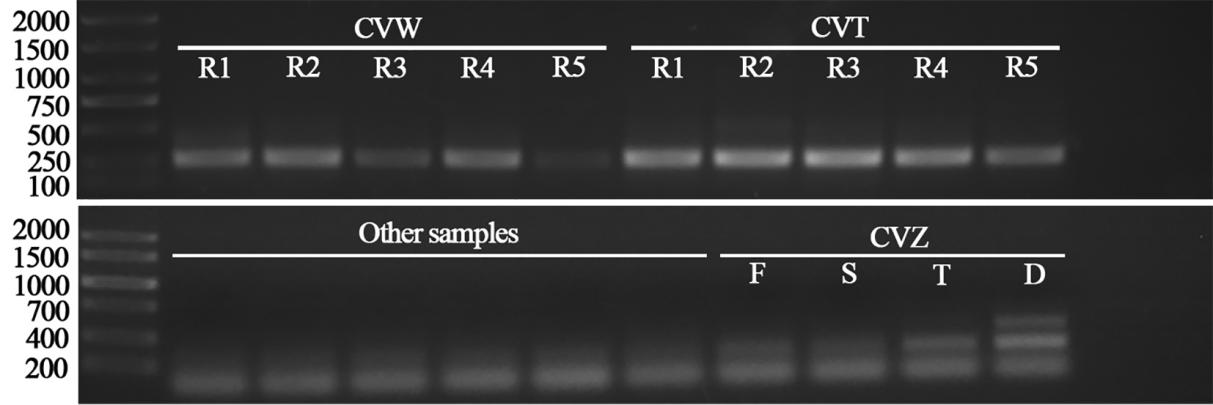


Figure S2-19. Expression analysis of *Cauliflower D*. Other samples refer to the materials unrelated to this study.


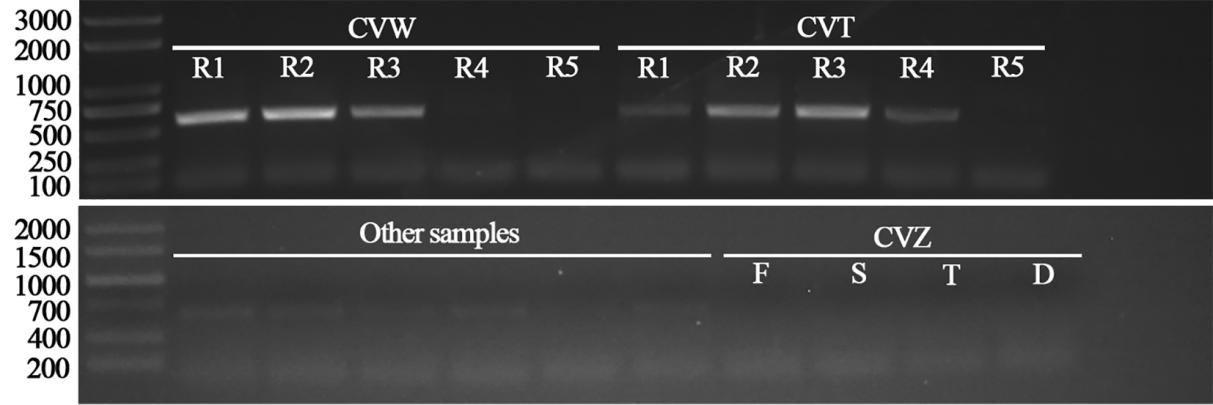


Figure S2-20. Expression analysis of *ARF1*. Other samples refer to the materials unrelated to this study.


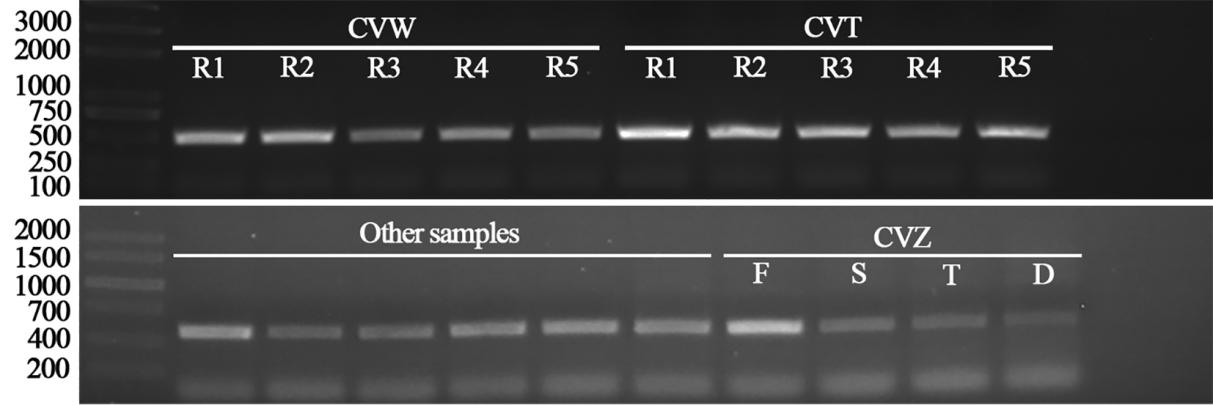


Figure S2-21. Expression analysis of *ARF2*. Other samples refer to the materials unrelated to this study.


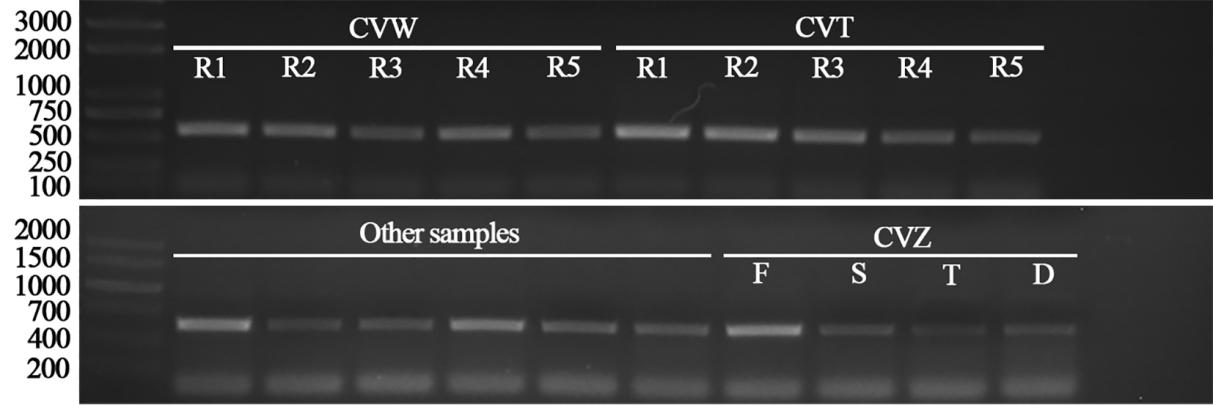


Figure S2-22. Expression analysis of *ARF3*. Other samples refer to the materials unrelated to this study.


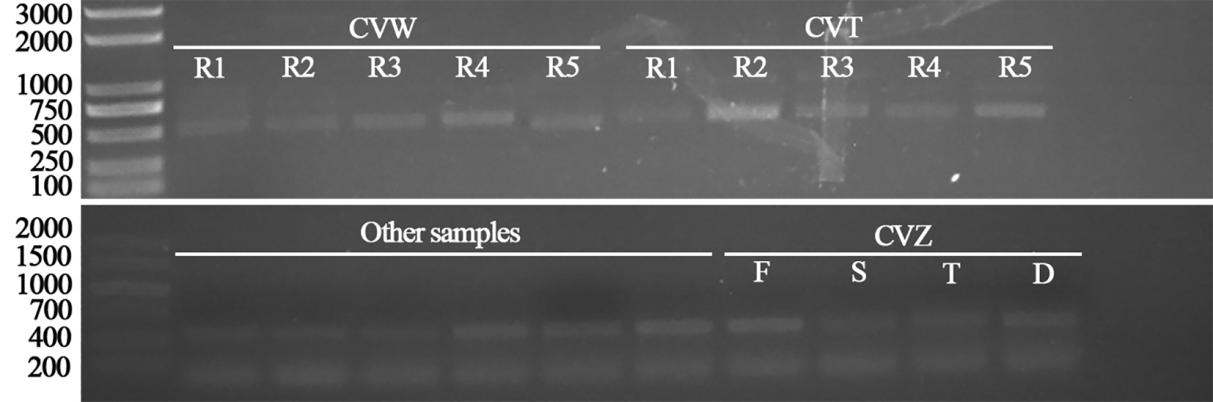


Figure S2-23. Expression analysis of *ARF5*. Other samples refer to the materials unrelated to this study.


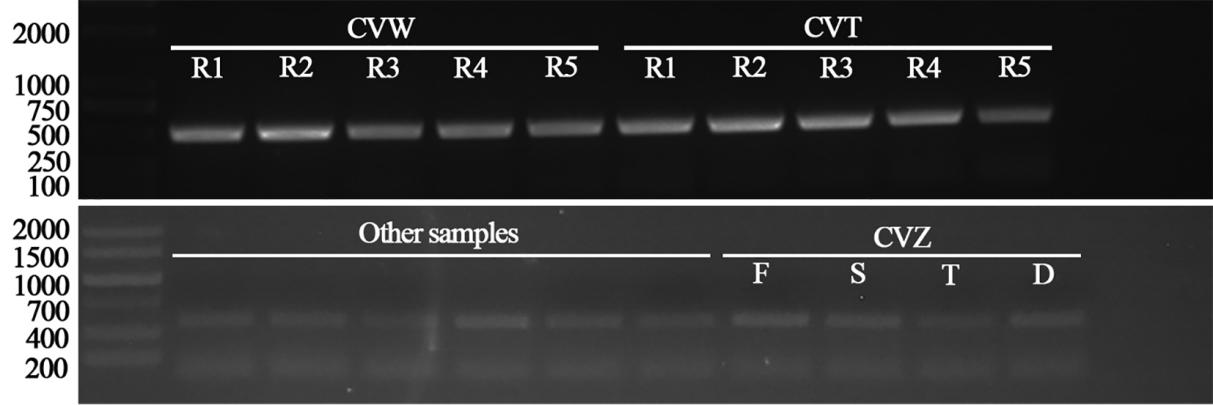


Figure S2-24. Expression analysis of *ARF6*. Other samples refer to the materials unrelated to this study.


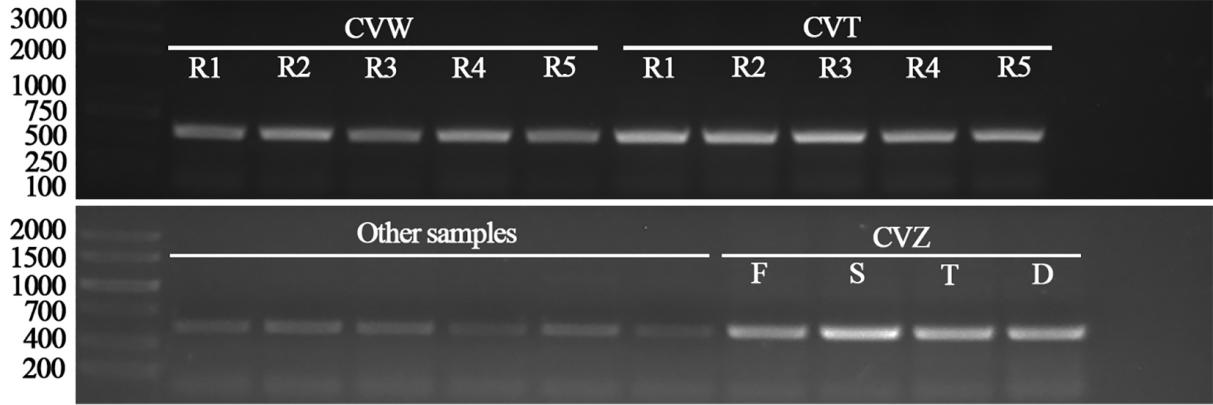


Figure S2-25. Expression analysis of *ARF8*. Other samples refer to the materials unrelated to this study.


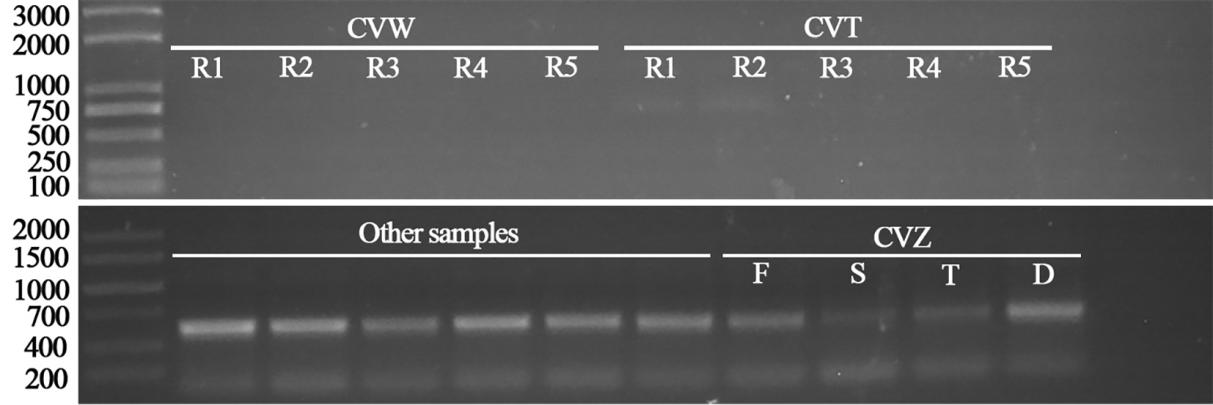


Figure S2-26. Expression analysis of *ARF9*. Other samples refer to the materials unrelated to this study.


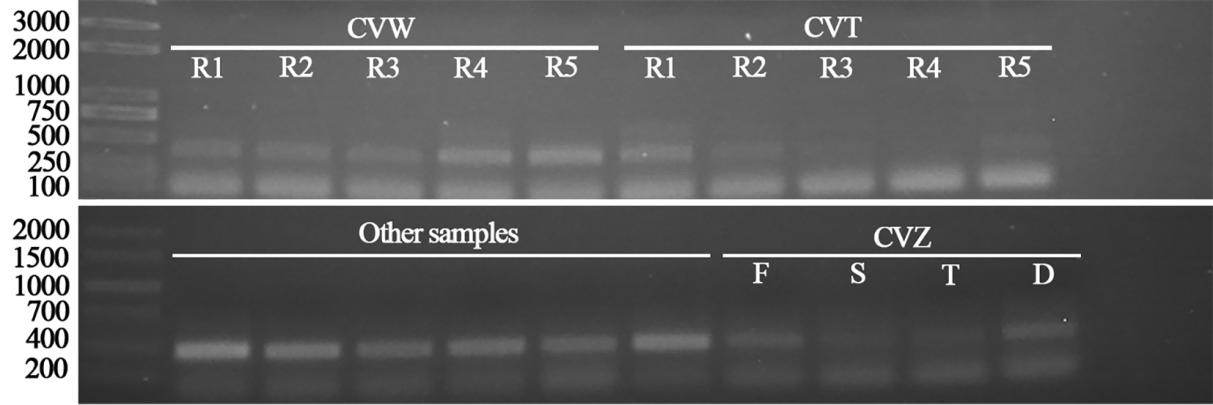


Figure S2-27. Expression analysis of *ARF11*. Other samples refer to the materials unrelated to this study.


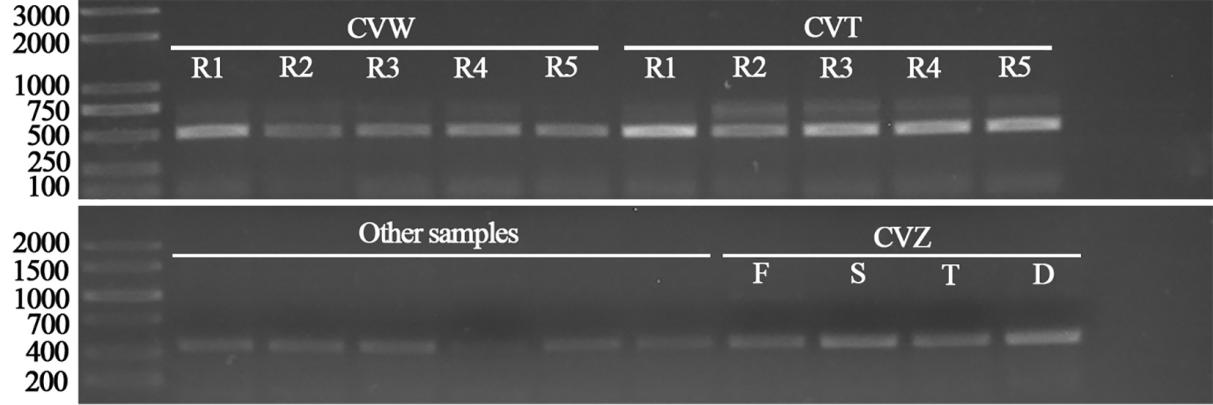


Figure S2-28. Expression analysis of *ARF18*. Other samples refer to the materials unrelated to this study.


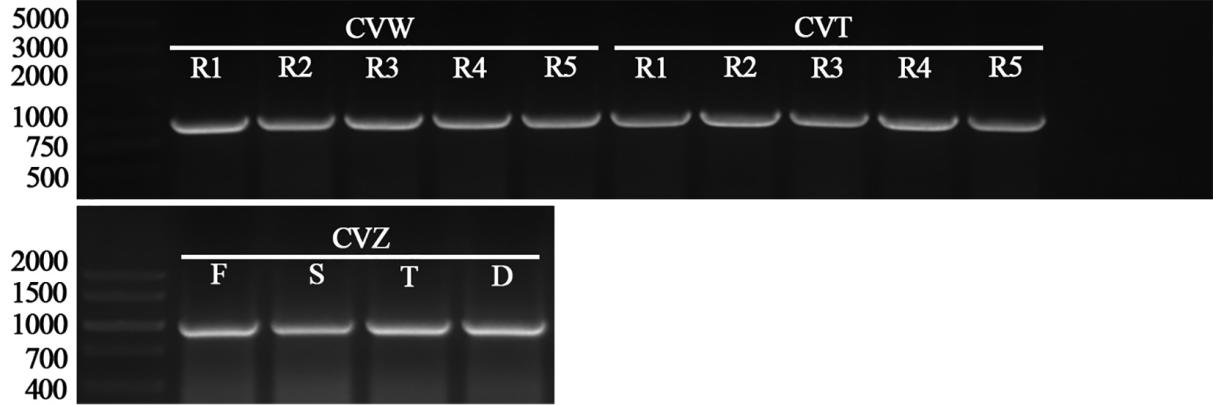


Figure S2-29. Expression analysis of *Actin*. Other samples refer to the materials unrelated to this study.
